# A high-resolution map of the Nile tilapia genome: a resource for studying cichlids and other percomorphs

**DOI:** 10.1186/1471-2164-13-222

**Published:** 2012-06-06

**Authors:** Richard Guyon, Michaelle Rakotomanga, Naoual Azzouzi, Jean Pierre Coutanceau, Celine Bonillo, Helena D’Cotta, Elodie Pepey, Lucile Soler, Marguerite Rodier-Goud, Angelique D’Hont, Matthew A Conte, Nikkie EM van Bers, David J Penman, Christophe Hitte, Richard PMA Crooijmans, Thomas D Kocher, Catherine Ozouf-Costaz, Jean Francois Baroiller, Francis Galibert

**Affiliations:** 1 Institut Génétique et Développement (UMR 6061) CNRS/Université de Rennes 1, Rennes, France; 2 Systématique, Adaptation, Evolution (UMR 7138) CNRS, Muséum National d'Histoire Naturelle, 75231, Paris, FRANCE; 3 INTREPID (INTensification Raisonnée et Ecologique pour une PIsciculture Durable) (UMR110) Cirad/Ifremer, 34398, Montpellier, France; 4 CIRAD, UMR AGAP, F-34398 Montpellier, France cedex, Montpellier, France; 5 Department of Biology, University of Maryland, College Park, MD 20742, Maryland, USA; 6 Animal Breeding and Genomics Centre, Wageningen University, Marijkeweg 40, Wageningen, 6709 PG, The Netherlands; 7 Institute of Aquaculture, University of Stirling, Stirling FK9 4LA, Scotland, UK; 8 UMR CNRS 6061, Faculté de Médecine, Université de Rennes 1, Rennes, France

## Abstract

**Background:**

The Nile tilapia (*Oreochromis niloticus*) is the second most farmed fish species worldwide. It is also an important model for studies of fish physiology, particularly because of its broad tolerance to an array of environments. It is a good model to study evolutionary mechanisms in vertebrates, because of its close relationship to haplochromine cichlids, which have undergone rapid speciation in East Africa. The existing genomic resources for Nile tilapia include a genetic map, BAC end sequences and ESTs, but comparative genome analysis and maps of quantitative trait loci (QTL) are still limited.

**Results:**

We have constructed a high-resolution radiation hybrid (RH) panel for the Nile tilapia and genotyped 1358 markers consisting of 850 genes, 82 markers corresponding to BAC end sequences, 154 microsatellites and 272 single nucleotide polymorphisms (SNPs). From these, 1296 markers could be associated in 81 RH groups, while 62 were not linked. The total size of the RH map is 34,084 cR_3500_ and 937,310 kb. It covers 88% of the entire genome with an estimated inter-marker distance of 742 Kb. Mapping of microsatellites enabled integration to the genetic map. We have merged LG8 and LG24 into a single linkage group, and confirmed that LG16-LG21 are also merged. The orientation and association of RH groups to each chromosome and LG was confirmed by chromosomal in situ hybridizations (FISH) of 55 BACs. Fifty RH groups were localized on the 22 chromosomes while 31 remained small orphan groups. Synteny relationships were determined between Nile tilapia, stickleback, medaka and pufferfish.

**Conclusion:**

The RH map and associated FISH map provide a valuable gene-ordered resource for gene mapping and QTL studies. All genetic linkage groups with their corresponding RH groups now have a corresponding chromosome which can be identified in the karyotype. Placement of conserved segments indicated that multiple inter-chromosomal rearrangements have occurred between Nile tilapia and the other model fishes. These maps represent a valuable resource for organizing the forthcoming genome sequence of Nile tilapia, and provide a foundation for evolutionary studies of East African cichlid fishes.

## Background

Tilapia is a common name for a large number of species belonging to the order Perciformes which accounts for one fourth of all vertebrate species. They are members of the family *Cichlidae* which consists of more than 3000 species distributed across tropical and subtropical regions. Tilapia are currently the second most farmed fish in the world with an annual production exceeding 2.8 million tons in 2010 [1]. Tilapia are a valuable source of protein for developing and emerging countries, but it is now also a prime fish commodity in developed countries. Apart from their domestic importance, there is a wealth of studies on different aspects of tilapia biology, e.g. on their physiology, endocrinology, immunology, toxicology and genetics. Tilapia have a short generation time, are sufficiently large in size for physiological studies and can be easily reared making them a perfect model system. They exhibit a versatile adaptability to different environmental conditions to match the vast array of their ecological habitats. They can tolerate incredible variations in temperature (12 to 43°C), pH (6 to 10), salinity (0 to 135 g/L), and oxygen levels (0.3 to 1.5 mg/L [2-5]). Therefore, they constitute exquisite models for environmental genomics, to analyse the interactions between the genome and the environment, and the adaptive responses to environmental stresses [6]. Because tilapia are closely related to the cichlid fishes in the Great Lakes of East Africa, which have undergone a spectacular radiation, they will contribute to our understanding of evolutionary mechanisms. The 2000 cichlid species in these lakes represent a collection of natural mutants that may provide insight into the genetic mechanisms of speciation and adaptation [7]. These unique biological features have motivated the development of a range of genomic tools for the Nile tilapia, *Oreochromis niloticus,* one of the most farmed tilapia species. An extensive collection of ESTs was recently constructed to aid the annotation of the forthcoming Nile tilapia genome and for gene expression studies [8]. Likewise the analysis of 106,259 BAC end sequences and their alignment on the genome sequence of four model fish species (stickleback, medaka, pufferfish and zebrafish) provides a valuable intermediate resource for the mapping of genes in cichlids [9]. The culmination of these efforts is the whole genome sequence currently being assembled by the Broad Institute (Cambridge, USA).

There are several economic traits in tilapia such as growth [10] and sex-ratio [11] that need improvement and require genetic markers for their selection. Likewise, identification of QTLs (Quantitative Trait Loci) for other economic traits are being performed in tilapia [12] as well as for immune responses [13]. Two Nile tilapia genetic maps were constructed, for QTL mapping and for selection purposes, of which the latest contains 538 microsatellites and 21 gene markers [14,15]. These genetic maps established 24 linkage groups although the tilapia karyotype is composed of just 22 chromosome pairs [16]. These genetic maps provided a first characterization of the tilapia genomes. Because only a few gene-based markers were mapped, synteny relationships with model fish species were only possible at low resolution. The map has been updated with a few more gene-based markers but the number of comparative markers remains limited [17,18].

Radiation hybrid (RH) mapping is suited to mapping all types of markers including gene-based markers, and can order them at high resolution. RH maps can integrate genetic maps through the mapping of polymorphic markers, as well as construct comparative maps through the mapping of non polymorphic markers (orthologous genes). In zebrafish the RH map allowed much higher gene-marker coverage of the genome and permitted comparisons with other vertebrates [19,20]. These advantages are particularly evident for fish species lacking genome tools, such as sea bass, where a gene-based RH map enabled comparisons with the genomes of stickleback, pufferfish, medaka and zebrafish [21]. Likewise, the two RH maps constructed for the seabream allowed comparisons with the pufferfish genome sequence [22,23]. Global synteny relationships were also established between three farmed Perciformes (seabream, European seabass and Nile tilapia) and with the model species (stickleback, medaka and pufferfish) [24].

High-resolution RH maps are also of great help in scaffolding genome sequences developed in shotgun projects [25,26]. In many cases draft and even “finished” genome sequences from shotgun projects contain large sequence gaps that imply inconsistencies in the placement of scaffolds. In addition, low in-depth sequences lack long-range continuity and provide only a fragmented view of a genome. This was precisely the case for the fugu genome sequence, which consists of 7213 unconnected scaffolds without any chromosome assignment [27]. The construction of RH panels for fish species has not been an easy task. To date, only four fish RH panels have been reported. Two zebrafish RH panels were derived from permanent cell lines [20,28]. More recently, a seabream RH panel was constructed from primary fibroblasts [22] and the European seabass RH panel was derived from splenocytes [21], thus avoiding the problems of genome rearrangements that arise in cell lines.

Here we describe the construction of a Nile tilapia RH panel derived from fresh splenocytes and a gene-rich RH map of 1358 markers. The RH map was integrated with the Nile tilapia karyotype by FISH analysis. This allowed us to assign the RH groups to the 22 chromosomes as well as to identify their orientation with respect to the centromere. The mapping of 154 microsatellites permitted the anchoring of the genetic map to the RH map. Amongst the different markers selected for the Nile tilapia RH map there was a large proportion related to growth and reproduction. In addition, a large number of SNPs identified in individuals from the 10^th^ generation of the widely cultured GIFT (Genetically Improved Farmed Tilapia) strain were included in the RH map (van Bers *at al.,* submitted to Molecular Ecology Resources).

## Results and Discussion

### RH panel

**Figure 1:**
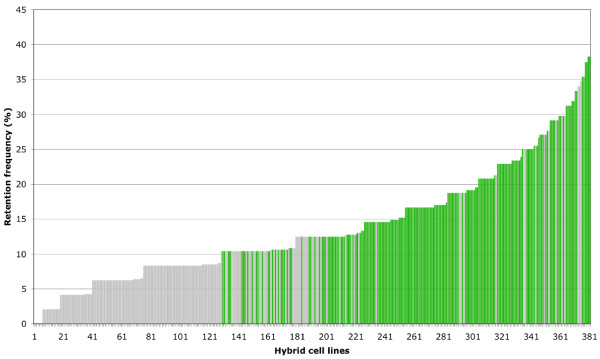
**Retention frequency of the Nile tilapia hybrid cell lines.** The hybrid cell lines are numbered from 1 to 381 on the X axis. Presence/absence of 48 microsatellite markers spread all over the tilapia genome [15] was estimated by PCR determination. Their retention frequency per clone is presented on the Y axis. The 190 hybrid cell lines selected on quantitative and qualitative criteria that constitutes the tilapia RH panel are in green.

We fused *Hprt*^—^ derivative CHO host cells with Nile tilapia splenocytes γ-irradiated at 3500 rad. Hybrid cells maintaining Nile tilapia chromosome fragments were selected based on their growth in HAT medium. A total of 381 hybrid cell lines were obtained through 3 fusion experiments. The retention frequency (i.e. the estimated percent of markers per clone) was determined for every clone by typing a set of 48 microsatellites selected from the genetic map [15]. We selected 190 hybrid cell lines on the basis of their retention frequency and their genome representation (Figure 1). Further typing of 56 additional markers on this 190 cell line panel led to a cumulative retention frequency of 11.7 %. This rather low retention value is compensated by the unusually high number of hybrid cell lines, which allowed us to substantially increase the number of genotyping data. The Nile tilapia RH panel was therefore constructed from fresh live cells that required no primary culture, an important condition to avoid genome rearrangements typical of permanent cell lines. Splenocytes are convenient to use, as they are abundant, their dissociation is easy and can be performed in a relatively short time preceding the irradiation step.

### Marker selection

**Figure 2:**
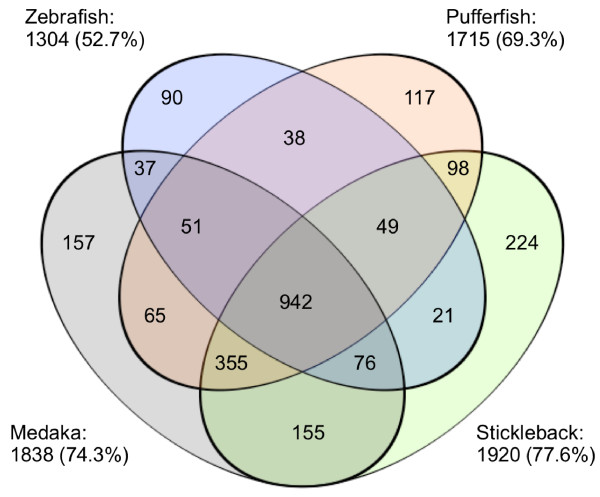
** Venn diagram representing the distribution of markers shared by Nile tilapia and stickleback/medaka/pufferfish/zebrafish.** Each model species is represented by an ellipse. Number of markers shared by two species or more are indicated in every intersection. For each model species, the number of markers and the percentage of the 2475 Nile tilapia markers are indicated.

A total of 16,195 Nile tilapia expressed sequence tags (ESTs) were collected, consisting of 5161 sequences from the CIRAD, 3537 sequences from the NCBI nucleotide database and 7497 sequences from the RBEST database (October 2008). A proportion of 3.5 % of these sequences were identified as simple repeats and masked by the RepeatMasker program. Sequence alignment with the CAP3 software resulted in 1476 contigs and 5692 singlets i.e. 7168 unique sequences putatively corresponding to as many genes (Additional file 1: Data S1). These 7168 sequences were aligned onto the stickleback, pufferfish, medaka and zebrafish genome sequences using the Exonerate software. A minimum score of 250, corresponding to a minimal alignment length of 50 bp, was applied following the usual recommendations to map orthologous ESTs [29]. A maximal alignment size of 300 bp was imposed to avoid hits that may in fact correspond to retrogenes rather than orthologs (retrogenes as opposed to pseudogenes which are characterized by non-sense or frame shift mutations). According to these criteria, 2475 of the unique sequences had a hit with at least one of the model genomes: 1920 (77.6%) had a hit with stickleback, 1836 (74.3%) with medaka, 1715 (69.3%) with pufferfish and 1304 (52.7%) with zebrafish (Figure 2). A total of 942 Nile tilapia sequences were conserved across all four model species, while 224 Nile tilapia sequences were conserved exclusively with stickleback, 157 with medaka, 117 with pufferfish and 90 with zebrafish.

We preferentially designed gene markers from Nile tilapia ESTs having the highest conservation with the stickleback. Although Nile tilapia is phylogenetically closer to medaka [30], the stickleback genome appears to be the best reference sequence because it is assembled with highest confidence [9]. Each marker was designed from the aligned region having the best homology (highest score) with the reference genome. This strategy minimized the possibility of an intron lying between the oligonucleotides. The Nile tilapia Illumina BeadArray contained 1536 markers consisting of 1300 genes, 97 BACs and 139 microsatellites. Sequence analysis of a Nile tilapia reduced representation library (RRL) resulted in the detection of 3569 SNPs. Of these, a subset of 384 SNPs was genotyped on the RH panel.

### Genotyping

Out of the initial 1536 selected markers and the subsequent 384 SNP markers genotyped on the RH panel, 1026 and 272 markers respectively showed an exploitable profile and were selected to construct the RH map. Roughly one third of the markers had to be removed from the initial set, as their calls could not be separated in two distinct clusters of presence and absence on the graphic representation. This ratio of failure is high, but is in the range of what was observed for our previous RH map constructions using PCR genotyping (dog, seabream and seabass). With this strategy a similar proportion of designed pairs of oligonucleotides either do not properly amplify the test DNA or amplify both the test DNA and the carrier hamster cell DNA and thus are not useful for RH map construction. The current Nile tilapia map was constructed with a final set of 1358 markers consisting of 850 genes, 154 microsatellites, 82 BACs and 272 SNP, including 60 markers genotyped by PCR (see the vector suite in Additional file 2: Data S2).

### RH group characteristics

The two-point analysis of the 1358 markers using the Multimap software started at a lod score of 4.0, which was then increased in a step-wise fashion up to a threshold of lod 7.0. The final map consists of 81 RH groups containing between 2 to 89 markers each for a total of 1296 markers. Sixty-two markers remained unlinked. The 1296 markers are spread in 1255 positions of which 1220 positions consist of a single marker, 32 positions contain two co-localized markers and three positions contain 3 to 6 co-localized markers. Multipoint analysis was carried out with CarthaGène software that ordered markers within each RH group and determined their distances expressed in centirays (cR_3500_). RH groups ranged in size from 5 to 1906 cR_3500._ Inter-marker distances vary between 1 and 164 cR_3500_ with an average of 27 cR_3500_. Characteristics of the RH groups in terms of size and marker content are presented in Additional file 3: S3. Assuming a Nile tilapia genome size of 1060 Mb [31] the mapping of 1358 markers corresponds to a density of 1.28 marker per megabase or one marker per 780 kb, when considering an even distribution of the markers.

The relationship between cR_3500_ and kilobase (kb) can be estimated from the ratio of RH to genetic distances (cR/cM) knowing the ratio between cM and kb. To this end, we identified 82 pairs of microsatellites separated by a known distance measured in genetic and RH units. The cumulated distance of these 82 couples is 18,446 cR_3500_ on the RH map and 604 cM on the genetic map i.e. a ratio of 30.5 cR_3500_/cM. The size of the Nile genome being 1060 Mb [31] and the size of the genetic map being 1311 cM the ratio was estimated to 840 kb/cM [15]. Therefore, the relation of physical unit to RH unit is estimated to be 27.5 kb/cR_3500_. The calculated size in kb of the RH map is 937,310 kb (34,084 cR x 27.5) and thus corresponds to a coverage of 88% of the entire genome size. Considering that ~96% of the markers (1298 markers out of 1358) were mapped in the 81 RH groups, one can estimate the probability of mapping a novel marker of interest in one of the existing RH group to be 96 %. This figure can most likely be considered as a better estimate of the coverage of the Nile RH map.

Because most of the markers designed for the Nile tilapia RH map were from ESTs, the genome regions that remain uncovered may correspond to gene-poor regions such as heterochromatin or regions containing genes that are poorly expressed.

### Integration of RH map and FISH data

**Figure 3:**
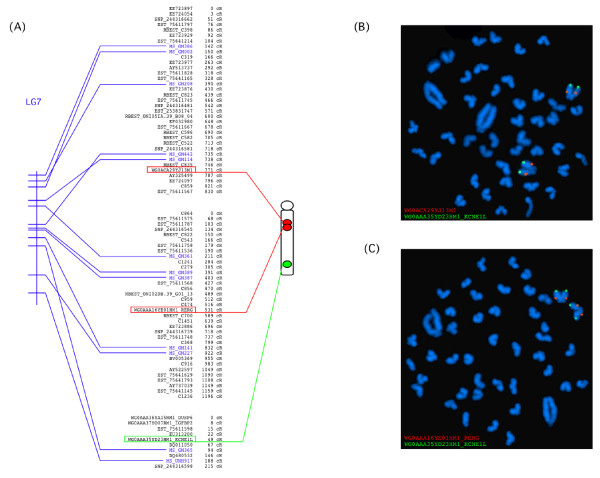
**(A) Integrated genetic-RH-FISH map of the tilapia chromosome LG7.** The RH map on the middle consists of three RH groups containing ordered markers whose coordinates are indicated in cR_3500_. Microsatellites (in blue) allowed the anchorage of the RH map to the genetic map [15] figured by a vertical bar on the left. Double-FISH of BAC clones highlighted by a red or green frame indicate the relative position of the RH groups on the chromosome symbolized on the right side. The chromosome is orientated with its centromere up. (**B**) Results of Double-FISH experiment of BAC clone WG0AAA35YD23HM1 revealed with FITC (green) and BAC clone WG0AAA16YE01HM1 revealed with Rhodamin (red) on a chromosome preparation. (**C**) Results of Double-FISH experiment of BAC clone WG0AAA35YD23HM1 revealed with FITC (green) and BAC clone WG0ACA29YJ13M1 revealed with Rhodamin (red) on a chromosome preparation.

The FISH mapping of BAC clones analyzed two by two allowed us (i) to assign RH groups to specific chromosomes with higher confidence, (ii) to orient them relative to each other and (iii) to localize centromeric and telomeric ends on the chromosome maps. They also served as a validation of linkage group assignment. In addition to ordering based on FISH mapping, the RH groups were tentatively ordered and orientated on the basis of the two-point analyses between markers close to RH group extremities and on the basis of the genetic map. The chromosome map of LG7 featuring RH and FISH maps along with the genetic map is presented in Figure 3. All chromosome maps are available in Additional file 4: Data S4 and online (http://www.BouillaBase.org). Table 1 presents chromosome characteristics in terms of number of markers and RH groups. We first selected 42 BAC clones that contained markers assigned to 33 RH. A second set of 48 BAC clones was selected from regions of interest based on synteny with reference species, obtained from the BouillaBase server.

**Table 1:** Characteristics of Nile tilapia chromosome maps

	**No of RH groups**	**Size (cR**_**3500**_**)**	**No. of positions**	**No of co-localized markers**	**No. of markers**	**No. of genes**	**No. of BAC**	**No. of microsat**	**No. of SNP**	**No. of anchors**			
										**Stickleback**	**Pufferfish**	**Medaka**	**Zebrafish**
**LG1**	2	1145	45		45	30	4	4	7	30	21	28	19
**LG2**	1	1038	44		44	34	3	2	5	34	24	26	23
**LG3**	5	1167	35	1	36	13	2	6	15	16	2	16	10
**LG4**	2	1296	55	2	57	45	2	3	7	42	23	38	28
**LG5**	3	1862	61	2	63	40	5	7	11	30	28	31	26
**LG6**	3	1622	60		60	43	4	8	5	44	24	30	32
**LG7**	3	2241	80		80	57	5	12	6	51	29	47	40
**LG** **8-24**	1	1677	61	2	63	48	2	3	10	42	26	40	35
**LG9**	2	1177	37		37	20	2	10	5	20	15	18	14
**LG10**	3	468	23		23	17	2	4	0	17	14	14	5
**LG11**	1	1465	48	3	51	29	5	7	10	27	19	21	19
**LG12**	1	1906	80	9	89	56	2	10	21	53	37	44	41
**LG13**	3	1349	48	2	50	31	2	4	13	34	29	30	26
**LG14**	1	1508	53	1	54	34	2	10	8	31	18	26	21
**LG15**	2	1269	47	3	50	36	2	5	7	29	20	28	25
**LG** **16-21**	4	1624	55		55	27	5	10	13	26	21	22	23
**LG17**	2	1489	51	3	54	31	3	7	13	25	16	24	23
**LG18**	2	1325	54	1	55	38	2	3	12	37	16	38	30
**LG19**	2	1462	53		53	36	3	6	8	36	27	30	24
**LG20**	3	1373	55	2	57	41	3	3	10	44	24	33	30
**LG22**	3	1095	40	4	44	25	2	4	13	24	8	19	19
**LG23**	1	1011	38	3	41	22	2	5	12	21	8	20	18
Sub-Total		30,569	1123	38	1161	753	64	133	211	713	449	623	531
Orphan groups		3515	133	4	137	71	13	8	45	72	38	57	54
Unlinked					60	31	5	8	16				
Total		34,084			1358	855	82	149	272				

Of the 90 initially selected BAC clones, FISH data from 45 BACs were not taken into account. They correspond to BACs producing many signals on different chromosome pairs (chimeric BACs or BACs that hybridize to duplicated regions), and/or to the observation of strong background signals in spite of the use of competitor and carrier DNA. They were deleted from the analysis and in many circumstances replaced by other BACs from the same linkage group. Successful hybridization results were those in which the two probes for the same LG group gave a clear signal and could be repeatedly observed in at least 10 metaphase spreads. Thus a total of 55 BAC markers (Table 2) were successfully hybridized and mapped in 39 RH groups. This allowed all of the main RH groups (n ≥ 30 markers) to have at least one BAC mapped by RH and FISH. For 17 chromosomes, two to four BACs were used to assemble several RH groups onto a single chromosome (Table 2). This was the case for instance, of LG7, constituted by three RH groups (Figure 3). Each of the 22 chromosomes can now be identified with the help of one to four fluorescent probes. This is a particularly important result because, with the exception of the largest chromosome pair (Chr1/LG3), which is three times larger than any other Nile tilapia chromosomes and of the second chromosome pair (Chr2/LG7), none of the other chromosomes can be easily distinguished using classical cytogenetic techniques, due to similarity in size and fluctuations in the chromatin condensation [32,33]. These BAC chromosome markers can also be used to identify orthologous chromosomal regions among closely related species within the Tilapia group (such as other *Oreochromis* spp., or *Sarotherodon*). They can also be used to provide insights on the evolution of chromosome regions that have taken place since the divergence of tilapia and other cichlids from their ancestors. The list of clones, their reference and chromosome assignation is presented in Table 2.

**Table 2:** BAC Markers positioned by RH mapping and FISH analysis

		**BAC**		
	**BAC Markers**	**Genoscope Name**	**384 Name**	**FPC Name**
**LG1**	WG0AAA14YI14RM1	WG0AAA14YI14	b03TI048I14	b03TI048BE07
	WG0AAA46YC14	WG0AAA46YC14	b03TI090C14	b03TI090BB07
	WG0AAA13YF01HM1	WG0AAA13YF01	b03TI047F01	b03TI047CC01
	WG0AAA42YA07HM1	WG0AAA42YA07	b03TI086A07	b03TI086AA04
**LG2**	WG0AAA30YG19HM1_ATRX	WG0AAA30YG19	b03TI074G19	b03TI074AD10
	WG0AAA2YH18HM1_FGF24	WG0AAA2YH18	b03TI032H18	b03TI032DD09
**LG3**	WG0AAA13YB11RM1	WG0AAA13YB11	b03TI047B11	b03TI047CA06
	WG0AAA36YM24RM1	WG0AAA36YM24	b03TI080M24	b03TI080BG12
**LG4**	WG0AAA11YA12	WG0AAA11YA12	b03TI045A12	b03TI045BA06
	WG0AAA22YF11HM1_LHX9	WG0AAA22YF11	b03TI066F11	b03TI066CC06
**LG5**	WG0ACA44YI02	WG0ACA44YI02	b03TI060I02	b04TI060BE01
	WG0AAA44YK23HM1_BAP	WG0AAA44YK23	b03TI088K23	b03TI088AF12
	WG0AAA44YP19RM1_LTPR1	WG0AAA44YP19	b03TI088P19	b03TI088CH10
	WG0AAA22YB14M1	WG0AAA22YB14	b03TI066B14	b03TI066DA07
**LG6**	WG0AAA45YN18RM1_TP53BP2	WG0AAA45YN18	b03TI089N18	b03TI089DG09
	WG0AAA34YG21HM1_MCM5	WG0AAA34YG21	b03TI078G21	b03TI078AD11
	WG0AAA31YE16HM1_ALDOA	WG0AAA31YE16	b03TI075E16	b03TI075BC08
**LG7**	WG0ACA29YJ13M1	WG0ACA29YJ13	b04TI039J13	b04TI039CE07
	WG0AAA16YE01HM1_RERG	WG0AAA16YE01	b03TI050E01	b03TI050AC01
	WG0AAA35YD23HM1_KCNE1L	WG0AAA35YD23	b03TI079D23	b03TI079CB12
**LG8-24**	WG0AAA42YA01RM1_KIR2.1_A	WG0AAA42YA01	b03TI086A01	b03TI086AA01
	WG0AAA33YD12	WG0AAA33YD12	b03TI077D12	b03TI077DB06
	WG0AAA46YD19	WG0AAA46YD19	b03TI090D19	b03TI090CB10
**LG9**	WG0AAA28YB24HM1_NPPC	WG0AAA28YB24	b03TI072B24	b03TI072DA12
	WG0AAA13YJ04M1	WG0AAA13YJ04	b03TI047J04	b03TI047DE02
**LG10**	WG0AAA38YC08HM1_TGFB3	WG0AAA38YC08	b03TI082C08	b03TI082BB04
	WG0AAA2YB24HM1_LOC485593	WG0AAA2YB24	b03TI032B24	b03TI032DA12
**LG11**	WG0AAA42YO20RM1_TGFB2R	WG0AAA42YO20	b03TI086O20	b03TI086BH10
	WG0AAA16YH17HM1_DLX3	WG0AAA16YH17	b03TI050H17	b03TI050CD09
	WG0ACA24YM03	WG0ACA24YM03	b04Ti034M03	b04TI034AG02
**LG12**	WG0AAA16YK18HM1_LIM6	WG0AAA16YK18	b03TI050K18	b03TI050BF09
	WG0ACA19YO21M1	WG0ACA19YO21	b04TI029O21	b04TI029AH11
**LG13**	WG0AAA41YB15RM1	WG0AAA41YB15	b03TI085B15	b03TI085CA08
	WG0ACA52YD05	WG0ACA52YD05	b04TI078d05	b04TI078CB03
	WG0AAA35YG16HM1_CLIC4	WG0AAA35YG16	b03TI079G16	b03TI079BD08
**LG14**	WG0AAA4YJ07HM1_DMRT1Y	WG0AAA4YJ07	b03TI034J07	b03TI034CE04
	WG0AAA30YO18HM1_CLDN13	WG0AAA30YO18	b03TI074O18	b03TI074BH09
**LG15**	WG0AAA47YB05M1	WG0AAA47YB05	b03TI091B05	b03TI091CA03
	WG0AAA29YA15HM1_FSHB	WG0AAA29YA15	b03TI073A15	b03TI073AA08
**LG16-21**	WG0ACA14YN04	WG0ACA14YN04	b04TI024N04	b04TI024DG02
	WG0ACA24YI10M1	WG0ACA24YI10	b04TI034I10	b04TI034BE05
	WG0AAA29YK07HM1_CLDN10C	WG0AAA29YK07	b03TI073K07	b03TI073AF04
	WG0AAA34YL09HM1_GDF6	WG0AAA34YL09	b03TI078L09	b03TI078CF05
**LG17**	WG0AAA28YI20RM1_BMP7	WG0AAA28YI20	b03TI072I20	b03TI072BE10
	WG0AAA1YC03RM1_APR_3	WG0AAA1YC03	b03TI031C03	b03TI031AB02
**LG18**	WG0AAA15YJ04M1	WG0AAA15YJ04	b03TI049J04	b03TI049DE02
	WG0AAA37YF19RM1_RAI2	WG0AAA37YF19	b03TI081F19	b03TI081CC10
**LG19**	WG0AAA33YH10RM1_NR5A2	WG0AAA33YH10	b03TI077H10	b03TI077DD05
	WG0AAA28YF18HM1_RAI17	WG0AAA28YF18	b03TI072F18	b03TI072DC09
**LG20**	WG0AAA32YO06RM1_GATA5	WG0AAA32YO06	b03TI076O06	b03TI076BH03
	WG0AAA30YN12HM1_CCA1	WG0AAA30YN12	b03TI074N12	b03TI074DG06
**LG22**	WG0AAA30YF08HM1_TGIF2LX	WG0AAA30YF08	b03TI074F08	b03TI074DC04
	WG0AAA12YB12RM1_LFI2	WG0AAA12YB12	b03TI046B12	b03TI046DA06
**LG23**	WG0AAA49YP19M1	WG0AAA49YP19	b03TI093P19	b03TI093CH10
	WG0AAA16YK10M1	WG0AAA16YK10	b03TI050K10	b03TI050BF05

All the BAC probes hybridized to the long arm of the chromosomes (such as LG7, shown in Figure 3) with the exception of LG15. This LG is composed of two RH groups and the BAC probes taken from each RH group both hybridized to the short arm of the chromosome. Chromosome LG15 is a small submetacentric chromosome in which the small arm is often clearly visible. It very probably corresponds to the chromosome 6 as defined by Ferreira et al. [34].

### Integration of RH and genetic maps

The published genetic map of Nile tilapia was constructed with 545 microsatellite markers and 20 gene markers present on 24 linkage groups (LG) [15]. The integration of the RH map onto this genetic map was established using the 132 microsatellites present in both maps. Hence, fifty of the 81 RH groups were connected to the 24 genetic linkage groups, placing them onto 22 chromosome maps which contained on average 2.3 RH groups per chromosome. These 50 RH groups totalize 1123 map positions containing 1161 markers, which represent 89.4% of the markers located on the map. The remaining 31 small RH groups containing two to nine markers totalize 137 markers, which correspond to 10.6% of the markers on the map. They cannot be assigned to any chromosomes presently. These groups ranged in size from 5 to 345 cR_3500_.

In the RH map we were able to associate two small LGs, LG8 and LG24 into a single chromosome by RH mapping of four microsatellite markers (GM027 and UNH129 from LG8, GM104 and GM173 from LG24). We also merged and confirmed by FISH analysis the previously grouped LG16 and LG21 into a unique chromosome [16]. LG2, LG11, LG12, LG14 and LG23 correspond each to a single RH group whereas 15 LGs are made of two to five RH groups (see Table 1). Consequently we have been able to locate the 24 linkage groups and placed them onto 22 chromosome pairs. For simplicity, we named the chromosomes maps after the genetic linkage groups (LG) of the Nile tilapia genome [15].

Overall the microsatellites in LG11, LG12, LG14 or LG23 are in the same order in the RH and genetic maps except for small local inversions that may be due to vector quality (see the data computation chapter) in one of the mapping methodologies. However, a larger discrepancy was observed in the lower part of LG14. This RH group was tentatively broken at higher lod scores (up to 7.0) but no reordering of the resulting groups was consistent with the genetic map. Given the high lod score to which this group stayed unbroken we believe that the correct order is that of the RH map.

### Comparative genomics

Synteny relationships were established from the markers of the assigned RH groups having localized orthologous genes in the sequences of model species. Orthologs localized in the “chromosome unknown” of model species were not taken into account. Of the 1123 mapped positions in the assigned RH groups, 277 markers allowed anchorage of the Nile tilapia genome with all four model fish species, 268 with three species, 165 with two species and 78 with one species representing a total of 788 orthologous markers providing 2320 anchors. Synteny relationships identified by two or more consecutive conserved markers defined a conserved segment (CS) while a single marker identified a singleton [35][36].

**Figure 4:**
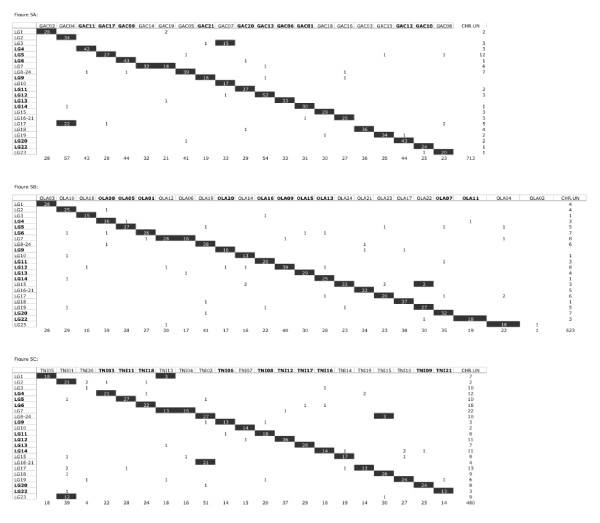
**Oxford grids between Nile tilapia and (A) stickleback, (B) medaka, (C) pufferfish.** Chromosomes are named as follows : LG : Nile tilapia chromosomes; GAC : stickleback chromosomes; OLA : medaka chromosomes; TNI : pufferfish chromosomes. Conserved chromosomes or conserved segments are figured in black squares containing the number of orthologous markers that identify them. Other numbers in the grid indicate the number of singletons. Chromosomes showing no synteny breakage between the four species are bolded.

The Oxford grid shown in Figure 4A recapitulates the CS found between Nile tilapia and stickleback. The Nile tilapia RH map and the stickleback genome sequence were connected by 713 anchors defining 23 CS. Seventeen CS correspond to entire chromosomes in which synteny is perfectly conserved between the two species. The Nile tilapia chromosome LG7 is made of two CS corresponding to stickleback chromosomes GAC14 and GAC19. Conversely, Nile tilapia chromosomes LG2 and LG17 consist of one CS each that are fused in stickleback to form chromosome GAC04. Furthermore, Nile tilapia chromosomes LG3 and LG10 both correspond to stickleback chromosome GAC07. This pattern of synteny would imply at least three inter-chromosomal rearrangements between the two lineages. The presence of two interstitial telomeric signals in Nile tilapia LG3 [32,33] suggests that this chromosome arose by two fusions. It has been suggested that these occurred within the cichlid lineage [34] but the stickleback-tilapia synteny (LG3 – GAC07) may suggest that these are older.

A total of 623 anchors identified 24 CS connecting the Nile tilapia RH map and the medaka genome sequence (Figure 4B). Synteny is entirely conserved between 20 chromosomes of the two species. As with stickleback, the Nile tilapia chromosome LG7 is made of two CS corresponding to medaka chromosomes OLA06 and OLA12. The Nile tilapia chromosome LG15 is also made of two CS, a large one corresponding to medaka chromosome OLA24 and a small one corresponding to medaka chromosome OLA22. This pattern implies two inter-chromosomal rearrangements that would have occurred in one or the other lineage. Medaka chromosome OLA02 is the only chromosome with no Nile tilapia chromosome counterpart in the Oxford grid (Figure 4B). However three arguments suggest that LG23 is the missing counterpart of medaka chromosome OLA02 in the Oxford grid: (a) a two-point analysis between end-markers links orphan group RH36 to LG23 (see Methods section) (b) RH36 and medaka chromosome OLA02 share five ortholog sequences (see Additional file 1: data S1) and (c) tilapia marker AF116240 located on LG23 has an ortholog sequence on medaka chromosome OLA02. The grouping of RH36 and LG23 creates an additional synteny breakpoint with medaka as well as with stickleback and pufferfish.

We identified 449 anchors connecting the Nile tilapia RH map and the pufferfish genome (Figure 4C) defining 24 CS between these two species. Synteny appeared totally conserved between 14 chromosomes of the two species. Chromosome LG1 corresponds to two CS, a large one that corresponds to chromosome TNI05 and a small CS made of two markers, which corresponds to chromosome TNI13. The chromosome LG7 consists of two CS, one with chromosome TNI13 and one with chromosome TNI04. Pufferfish chromosome TNI01 is made of two CS corresponding to LG2 and LG23 respectively. Chromosome TNI02 is also made of two CS with Nile tilapia chromosomes LG8-24 and LG16-21. A small additional segment of chromosome TNI15 is conserved with Nile tilapia chromosome LG8-24. This pattern of conservation implies four inter-chromosomal events. Assignation of the orphan group RH36 to LG23 as discussed above would create an additional CS and would imply another inter-chromosomal event. Additional CS exist but have not been identified yet such as in chromosome LG3 for which most of the pufferfish orthologs are located in the “chromosome unknown” file of the pufferfish assembly.

Ten chromosomes show no synteny breakage across the four species (bolded in Figure 4A, 4B, 4C). Twenty five singletons were identified between Nile tilapia and stickleback, 44 between Nile tilapia and medaka and 38 between Nile tilapia and pufferfish. These singletons suggest putative new CS but they also may be artefacts. Indeed the orthologous location of a given gene in a model species was defined as the best hit on the genome sequence of that species. However the best hit may in some instances have corresponded to a paralog especially when the true ortholog has been lost as hypothesized by Soler et al. [9] to explain a possibly overestimated number of breakpoints. Consequently each singleton will have to be established as a new CS by the mapping of additional and informative markers.

The number of CS appeared similar between Nile tilapia and each of the three reference models investigated in this work. This is in concordance with what was previously observed in the comparative map of the sea bass genome and the same fish models [21]. Finally, Nile tilapia and zebrafish were considered too distant phylogenetically to establish a pattern of chromosomal conservation despite the fact that 531 anchors were identified between the two species.

**Figure 5:**
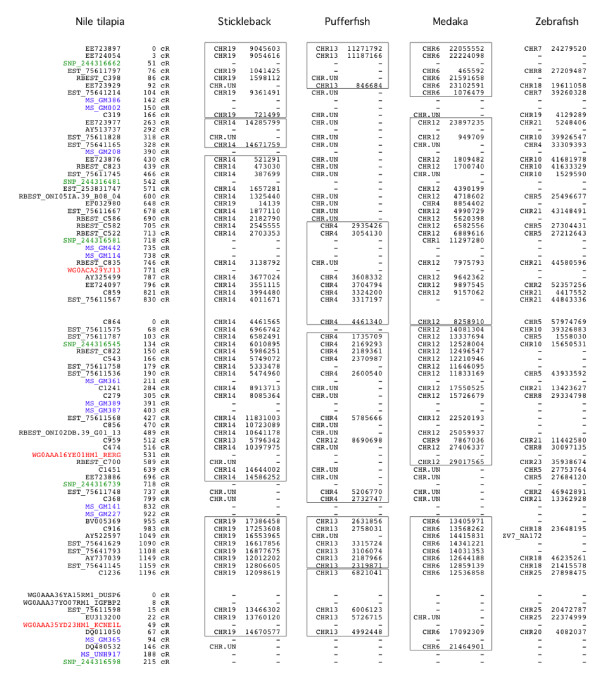
**Comparative map of the Nile tilapia chromosome LG7.** Column 1 corresponds to marker names. All markers are gene-based markers except (**a**) those with prefix “MS” which correspond to microsatellites (in blue) taken from Lee et al. (2005), (**b**) those with prefix “WG0” which are BAC end markers (in red) and (**c**) those with prefix “SNP” which correspond to SNP-based markers (in green). Column 2 corresponds to marker coordinates expressed in centiRays (cR_3500_). Following columns correspond to comparative data with, from left to right, stickleback, pufferfish, medaka, zebrafish. For every marker, chromosome numbers and coordinates of the putative orthologs in the genome sequences of the four model species are displayed. CSO between Nile tilapia and stickleback/medaka/pufferfish are figured in boxes.

On an intra-chromosomal scale, Conserved Segments Ordered (CSO) are regions in which the order of orthologous genes is perfectly conserved [35,36]. The simultaneous comparison of several species allowed us to ascertain the extent and boundaries of shared CSO while also revealing the breakpoints that arose in some lineages. The Nile tilapia RH map aimed at identifying these CSO with stickleback, medaka and pufferfish in order to benefit from the comprehensive sequencing of these model genomes. Thus the location on the Nile tilapia genome of unmapped genes having a clear orthologous relationship with genes of the model species could be hypothesized with high confidence. CSO between stickleback, medaka, pufferfish and Nile tilapia were identified using the AutoGRAPH web server and are presented in Table 3. Detailed CSO of LG7 are shown in Figure 5. Comparative maps of each of the 22 chromosomes are presented in Additional file 5: Data S5.

**Table 3:** Syntheny relationships identified with the genomes of model fish species

**Tilapia**	**Stickleback**		**Medaka**		**Pufferfish**	
	**CS**	**CSO**	**CS**	**CSO**	**CS**	**CSO**
**LG1**	1	4	1	2	2	2
**LG2**	1	3	1	1	1	3
**LG3**	1	1	1	3	un	un
**LG4**	1	7	1	4	1	3
**LG5**	1	5	1	5	1	5
**LG6**	1	5	1	5	1	5
**LG7**	2	4	2	4	2	5
**LG8-24**	1	5	1	5	2	4
**LG9**	1	2	1	2	1	2
**LG10**	1	2	1	1	1	1
**LG11**	1	6	1	3	1	5
**LG12**	1	4	1	2	1	5
**LG13**	1	2	1	2	1	2
**LG14**	1	1	1	2	1	2
**LG15**	1	3	2	6	1	2
**LG16-21**	1	4	1	5	1	2
**LG17**	1	4	1	3	1	1
**LG18**	1	6	1	6	1	2
**LG19**	1	5	1	4	1	2
**LG20**	1	7	1	3	1	1
**LG22**	1	4	1	5	1	2
**LG23**	1	5	1	5	1	1
**All chr**	**23**	**89**	**24**	**78**	**24**	**57**

CS: Conserved Segments.

CSO: Conserved Segment Ordered.

We identified 90 CSO between Nile tilapia and stickleback. Chromosomes LG14, LG9, LG10 and LG13 underwent few rearrangements with one to two CSO only (1, 2, 2, 2 respectively) while for LG4, LG18 and LG20, seven, six and seven CSO respectively were identified, showing evidence of considerable rearrangement. A total of 79 CSO were identified between Nile tilapia and medaka. One CSO was detected in chromosomes LG2 and LG10 while LG15 and LG18 were the most rearranged. Only 57 CSO were detected between Nile tilapia and pufferfish (Table 3).

A higher number of CSO was identified with stickleback than with medaka and pufferfish. Considering that the phylogenetic position based on various parameters between Nile tilapia and the other model species indicates a closer relationship between Nile tilapia and medaka one would rather have expected the contrary [30,37]. However, in terms of sequence similarity Nile tilapia is actually closer to stickleback than to medaka and pufferfish. This apparent discrepancy, if confirmed, would indicate that forces shaping the overall genome structure are different from those affecting gene function and evolution. Finally, from a technical standpoint, it is important to recall that the number of observed CSO shared by two species depends in part on the number of anchors used to establish the respective comparative maps. Indeed the higher the number of orthologous genes is, the more resolution the comparative map will have. Ideally in this study, the comparative maps should be based solely on 1:1:1:1 orthologs between Nile tilapia, stickleback, medaka and pufferfish. However only half of the markers satisfy this condition. Comparative maps based on this smaller number of markers have too little resolution to reach any conclusions.

The sequence assembly status in some genomic regions of model species also prevents the identification of CSO with Nile tilapia. For example, assemblies of stickleback GAC17 (syntenic of Nile tilapia LG5), medaka OLA09 (syntenic of LG12), pufferfish TNI04 (syntenic of LG7) or TNI19 (syntenic of LG17) are incomplete and lead to an underestimation of the number of CSO. Assuming a conserved gene order with the Nile tilapia the RH map would provide an opportunity to locate the unassigned contigs of model species. In this way the Nile tilapia RH map can be seen as a tool for improving the sequence assemblies of other fish species.

Chromosomes LG10, LG14, LG9 and LG13 appear to be the least rearranged between Nile tilapia and the three model genomes. Conversely LG5, LG6 and LG18 were the most rearranged. This observation suggests that the genome plasticity and the underlying evolutionary constraints are not evenly distributed across the genome.

## Conclusions

Through a spectacular decrease in cost and with the capability to generate more than hundred gigabases per week, the New Sequencing Technologies (NGS) have revolutionized the field of genomics over the last few years. It is now possible to obtain deep knowledge of the genomes of many more species than we could have dreamed of even ten years ago. However the main drawback of NGS is the short length of their reads. Although steadily increasing, sequence reads are still very short (~100 nucleotides). This is not a problem when the goal is to re-sequence individuals and align the reads to a reference sequence. However this short size, even with a pair ends sequencing approach, renders the problem of *de novo* sequencing of large genomes difficult. Many of the novel assemblies produced with this approach are composed of a very large number of scaffolds [38]. This discontinuity does not affect gene discovery, polymorphism analysis and sequence comparison between closely related species but it greatly limits the study of the genome structure and evolution. RH mapping and FISH mapping of markers present in different contigs and scaffolds allow to link them and deduce larger super scaffolds.

Here we report the construction of a high-resolution RH map of Nile tilapia containing ESTs, genes, microsatellites and SNPs. The RH map has an estimated density of one marker every 780 kb. Fifty RH groups, which contained the vast majority of the markers (1161 out of 1358), were assigned to the 24 previously known LGs, which in turn were located and oriented on the 22 Nile tilapia chromosomes through BAC multicolor FISH mapping on metaphase chromosomes. Already this RH map allows us to locate a large number of physiologically important genes. For example, group RH17 located on chromosome LG15 contains the estrogen receptor gene together with *gata4* and the follicle-stimulating hormone gene (*fshb*). This last gene has been shown to regulate the activity of Gata4, a transcription factor involved in ovarian function, by regulating the aromatase *cyp19* gene [39]. Likewise, the growth hormone receptor gene *ghr1* (marker C456) was mapped in the group RH3 assigned to chromosome LG12 and the growth hormone receptor gene ghr2 (marker C474) was mapped in the group RH9 assigned to chromosome LG7.

The RH map associated to the FISH data also offers a detailed synteny analysis with three of the four model species (stickleback, medaka, pufferfish). Due to the great evolutionary distance separating Nile tilapia from zebrafish (>300MY), it was not possible to reach definitive conclusions about synteny between these species. Furthermore, it provides a foundation for studying karyotypic evolution in the flocks of haplochromine cichlids in East Africa, including the evolution of sex chromosomes [40-42] and the origins of B chromosomes [43]. By contributing to the construction of a golden path for the Nile tilapia genome assembly, these maps will enable QTL and association mapping of adaptive traits in each of the haplochromine species flocks.

The mapping of a number of SNPs derived from 20 individuals of the 10^th^ generation of the widely cultured GIFT strain are included. To the best of our knowledge these SNPs are the first set of genome wide SNPs publicly available for Nile tilapia. SNPs are gaining popularity for use in e.g. parental assignment [44] and for the estimation of genetic parameters in tilapia breeding. The 272 SNP markers were shown to allow the discrimination between different strains and species of tilapia (van Bers *at al.,* submitted Molecular Ecology Resources), and will be used in the near future to assess the genetic diversity of natural populations of Nile tilapia (Richard Crooijmans, personal comm.). The physical mapping position of these SNPs determined in this study allows the selection of unlinked SNPs for these future applications. Finally the map will help to place and orientate on the Nile tilapia karyotype many of the scaffolds identified in the forthcoming tilapia genome sequence determined with the Illumina technology and assembled by the BROAD Institute.

## Methods

### Construction of a Nile tilapia radiation hybrid panel

The RH map was constructed from a fully-inbred homozygous clonal line of *O. niloticus* consisting of all-female fish, generated at the University of Stirling (Scotland, UK). These fish were derived by gynogenesis from a strain originating from Lake Manzala (Egypt) [45]. A panel of radiation hybrid cell lines was constructed using the methodology described previously [22,46]. Briefly for each fusion, a splenocyte suspension was prepared using one clonal fish as described in Guyon et al. [21]. The suspension was γ-irradiated at 3500 rad. Splenocytes were fused with *Hprt*^—^ derivative CHO cells in a 5: 1 ratio (Splenocyte/CHO) in the presence of polyethylene glycol 1500 (Roche, Mannheim, Germany). Cells were seeded in 6-well microplates at a total concentration of 150,000 cells per well and cultivated with HAT medium for 3 to 4 weeks until hybrid clone appearance. Each clone was recovered and further cultivated under HAT selection approximately one week in a 60 mm diameter Petri dish. After trypsinisation DNA was extracted from individual clones using the NucleoSpin Tissue kit (Macherey-Nagel, Düren, Germany). DNA concentration was estimated by fluorescence quantitation using Quant-iT Picogreen assay kit and a Qubit measuring device (Invitrogen, Carlsbad NM, USA). DNA extracts of hybrid cell lines were amplified by a Whole Genome Amplification (WGA) procedure when additional material was needed. In these cases, two separate WGA were performed with 10 ng of DNA each using V2 GenomiPhi kits (GE healthcare, Fairfield CT, USA). WGA products were pooled providing ~10 μg of material for subsequent genotyping. The reliability of WGA was previously demonstrated by Senger et al. [22]. Fishes were anesthetized with the addition of phenoxybarbtal in the water tank (3ml per 10 liter) and then sacrificed following the rules established by the ethical local committee.

### Marker selection

Nile tilapia expressed sequences (ESTs and complete mRNA sequences) were either downloaded from NCBI nucleotide and RBEST (http://reprobio.nibb.ac.jp/) databases or provided by the CIRAD (Montpellier, France). After masking simple repeats using the RepeatMasker program [47] sequences were aligned together using the CAP3 software [48] with default parameters. The resulting unique sequences were aligned on stickleback (v1.0 assembly), pufferfish (v8 assembly), medaka (v1.0 assembly) and zebrafish (Zv7 assembly) genome sequences using the Exonerate software [49]. Orthologous sequences were searched using a minimal score of 250 and an alignment size of 80 to 300 nt. Sequences which showed conservation with the highest number of species were selected to design markers. For every marker, the coordinate of the best hit on each of the model genomes was considered as the location of the putative ortholog in the model species. In addition, BAC end sequences from the CIRAD and the University of Maryland (USA) [9] containing genes of interest were selected for the RH mapping. Microsatellites of the genetic linkage map of Nile tilapia (second generation) [15] were also selected in order to anchor the genetic map to the RH map.

In addition SNP markers were identified in a pool of 20 individuals obtained from the 10^th^ generation of the GIFT population (WorldFish Center, Malaysia). The development of the SNP markers is described in detail elsewhere (van Bers et al., in prep). In brief, an RRL was prepared and sequenced using the Illumina GAI sequencing technology. Pooled DNA was digested with the *RsaI* restriction enzyme and fragments of 3.5-4 kb were isolated by electrophoresis. The fragments were sheared and used for high throughput sequencing. Sequence reads stringently filtered for quality were first assembled using SSAKE [50] to constitute a reference draft sequence. Less stringently filtered reads were subsequently mapped onto the reference draft using MAQ [51], allowing the detection of SNPs. The Minor Allele Frequency (MAF) was calculated based on how many times a SNP was observed in the sequence data. Only SNPs showing the minor allele at least three times were considered as true SNPs. Illumina type II SNPs with a design score >0.75 and a MAF >0.16 were selected for genotyping.

Marker sequences used to construct the map are given as supplemental Additional file 6: data S6 and have been submitted to NCBI: accession numbers, ss 244316446–244316740 (SNP markers) and 253831740–253831804 and 75611463–75642120 (EST markers). BAC markers are deposited in http://www.BouillaBase.org.

### Genotyping

Genes, microsatellites and BAC end markers were typed on the Nile tilapia RH panel using the 1536-marker Illumina BeadArrays system. Amplification of 45–55 bp loci was performed using oligonucleotides 20–23 nt in length complementary to the Nile tilapia sequences and designed using the Illumina proprietary design program. For this, the program was adapted to design oligonucleotides flanking non-polymorphic sites. Oligonucleotides were synthesized and spotted onto two 96-sample array matrices by the Illumina company (San Diego, CA, USA). The genotyping was carried out using the Illumina GoldenGate technology. SNP markers were typed on the RH panel with a 384 SNP multiplex genotyping assay using the GoldenGate Assay. Oligonucleotides were designed flanking a SNP according to the Illumina design program specifications. The assay was deployed on a BeadXpress platform using the Veracode technology.

**Figure 6:**
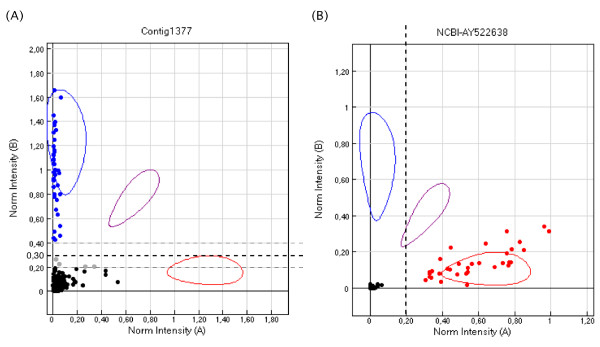
**Cartesian plots of radiation hybrid typing by the GoldenGate technology.** (**A**) Dots located above a threshold of 0.30 on the y-axis corresponded to positive clones scored “1”, dots located under the threshold corresponded to negative clones scored “0”. Dots located close to the threshold were considered as ambiguous results scored “2” (grey dots). (**B**) According to the overall repartition of dots on the profile of typing the threshold was lowered to 0.20 on the x-axis.

The Illumina Genome Studio software was used to visualize typing results and score the presence/absence of the markers in the hybrid cell lines by a method adapted from McKay et al. [52]. In our experiment all markers including the SNPs behaved as homozygous markers because the hybrid lines were constructed from a homozygous clonal Nile tilapia line implying homozygosity at all loci. Consequently a single allele-specific oligonucleotide was used in the GoldenGate extension step and only the axis of the corresponding fluorochrome was taken into account for a given marker. Clones located above a threshold of 0.3 on this axis on the cartesian plot were scored as present regardless to the value on the other axis. Clones located under the threshold were scored as absent. The threshold was adjusted depending on the overall distribution of dots on the typing profile. Clones located close to the threshold were scored as ambiguous (Figure 6). Microsatellites used to characterize hybrid cell lines and additional BAC end markers were typed by PCR and scored as described in Guyon et al. [21].

#### Data computation

All vectors were integrated in a single file and a two-point analysis was performed using the Multimap v2.0 software [36] starting at a lod score of 4.0. The multipoint analysis was performed with the CarthaGène v1.0 software [53]. RH groups that harboured obvious aberrations were re-analysed at higher two-point lod scores of up to 7.0 before performing the multipoint analysis again. Distances between markers were expressed in centirays (cR_3500_). Coordinates of the putative orthologous genes in the four model genomes were aligned with the corresponding Nile tilapia markers on the graphic representation. Ordered RH groups were tentatively oriented according to two-point lod scores between their end-markers. Similarly, comparison of the lod values obtained between the markers at the extremities of two RH groups, which are for other reasons supposed to be close to one another on the genome allowed to confirm or rule out this hypothesis. The microsatellite order on the RH map was compared with that of the linkage map [15]. Conserved Segments (CS) and Conserved Segments Ordered (CSO) [35,36] between Nile tilapia and the model species were identified using the AutoGRAPH web server [54].

### CMap construction

A comparative map viewer was constructed using the web-based tool CMap [55] in order to visualize and compare the RH map with the genetic map of *O. niloticus*[14,15]. Tab-delimited map and correspondence files were created between the two maps based on marker names and loaded into the CMap database. In addition, comparative maps were created between each of the RH map, the *O. niloticus* genetic map, as well as maps of two haplochromine cichlid lineages: *Astatotilapia burtoni*[56] and *Metriaclima zebra/Labeotropheus fuelleborni*[57]. The comparative maps can be viewed through CMap at http://cichlid.umd.edu/cgi-bin/cmap/viewer.

### Fluorescent In Situ Hybridization (FISH)

In order to validate the anchoring of the RH groups to a particular linkage group and to orient correctly the groups on the corresponding chromosome, physical mapping was performed by fluorescent in situ hybridization (FISH) using as probes at least two BACs per linkage group/chromosome.

#### Chromosome preparations

Chromosomes were prepared by direct in vivo methods from spleen and head-kidney cell suspensions as described in Fischer et al. [58], with a hypotonic treatment performed in a 28°C water bath for only 20 min. All chromosomes preparations were made from the XX genotype of the sequenced strain.

#### BAC clones preparation and purification

BAC DNA were purified from two Nile tilapia BAC libraries, T3 library (mean insert size 145 kb) and T4 library (mean insert size 194 kb) [59]. Individual clones were cultured in 100 ml 2YT broth with 12.5 μg/ml chloramphenicol at 37°C for 24 hours. BAC DNA was isolated using the plasmid midi kit (Qiagen) following the manufacturer’s protocol, obtaining between 20 to 50 μg yields. The BAC DNA was then validated and its quality verified before FISH by PCR using specific primers.

#### Fluorescence in situ hybridization

pt?>BAC probes were prepared from 2 μg of BAC preparation fragmented by heating at 98°C for 30 min and subsequently labelled with either DIG- or biotin using a High Prime DNA labelling kit (Roche Applied Science), according to the supplier’s protocol. To facilitate double BAC FISH experiments, we first prepared stock solutions for each component which were stored separately at −20°C. The BAC probe pellet was resuspended in the hybridization buffer (50% formamide, 2xSSC, 10% dextran sulphate and 50 mM of sodium phosphate) at a concentration of 16 ng/μl and incubated overnight at 37°C under constant agitation. The competitor consisted of sonicated *O. niloticus* DNA and the carrier was sonicated bovine DNA. Both were resuspended in hybridization buffer at 8 μg/μl and 10 μg/μl, respectively.

For the FISH, 2.5 μl of BAC probe, 1 μl of competitor and 4 μl of carrier were preheated at 45°C in a water bath before mixing. The BAC probe mixture was then denatured for 5 min at 85°C and pre-hybridized in a water bath at 37°C for 90 min to eliminate non-specific signals generated by small abundant repetitive sequences (essentially microsatellites) present in BAC inserts or generated by the BAC vector. For the double FISH, both BAC probes were pooled, mixed just *prior* to the hybridization. Chromosomes on slide preparations were denatured for 10 seconds in 70 % formamide/2x SSC at 72°C, followed by a dehydration in 70 %, 80 %, 98 % ethanol bath series. After quickly air drying the slides, the reannealed probe mixture was loaded onto the slides, covered with 22 x 22 mm plastic coverslips, and hybridized at 37°C in a moist chamber during 48 h. For the post-hybridization, the coverslip was removed and the slides were washed in 0,4x SSC, 0,3% Tween 20 (v/v) at 60°C for 2 min and 2x SSC, 0.1% Tween 20 (v/v) at room temperature for 1 min. The hybridized probes were detected with 30 μl of a dual colour solution of anti-dig Rhodamin/streptavidin-FITC (Roche Diagnostics) placed under a 24 x 40 mm coverslip, during 5 min in the dark. Slides were then washed three times in 4x SSC, 0.1% Tween 20, 2 min each followed by dehydration in a series of increasing ethanol percentages.

For the FISH observations, the slides were mounted in DAPI/antifade and analysed with a fluorescent microscope Zeiss Axio imager M1 equipped with a CoolSNAP camera (Photometrics) and the animal karyotyping/FISH imaging software *Genus* (Genetix).

## Competing interest

The authors declare no competing interest

## Authors’ contributions

FG, JFB, COC, HDC and TDK conceived the experiments. DJP selected the clonal line of *O. niloticus.* RG selected the gene markers, constructed the RH map. MR and NA made the RH panel and genotyped the gene markers. NEMVB and RPMC identified and genotyped the SNP markers. LS and MC compared the genetic and RH maps. HDC, CB and EP selected and prepared the BACs. JPC, CB, ADH, MRG and COC made the FISH experiments. FG and RG wrote the manuscript. All authors read and edited the manuscript.

## Supplementary Material

Additional file 1 **Data S1.** List of the 237 gene markers derived from contigs made with CAP3 software using fish ESTs from NCBI, RBEST and CIRAD as listed under the name of each marker. Click here for file

Additional file 2 **Data S2.** RH vector suite. Click here for file

Additional file 3 **Data S3.** Table of RH group characteristics. Click here for file

Additional file 4 **Data S4.** Integrated maps of the 22 chromosomes. The legend of Figure 3A applies to each of the 22 chromosome figures. Click here for file

Additional file 5 ** Data S5.** Comparative maps of the 22 chromosomes. The legend of Figure 5 applies to this file. The order of the columns is the same as in Figure 5. Click here for file

Additional file 6 Names and nucleotide sequences of markers located on the RH map. Click here for file
