# Supplementary material for: A high-resolution map of the Nile tilapia genome: a resource for studying cichlids and other percomorphs
Source: BMC Genomics. 2012 Jun 6;13:222. doi: 10.1186/1471-2164-13-222 (PMC3441813; doi:10.1186/1471-2164-13-222)
Supplement: Additional file 4 — Data S4. Integrated maps of the 22 chromosomes. The legend of Figure 3A applies to each of the 22 chromosome figures. [file 1471-2164-13-222-S4.pdf]

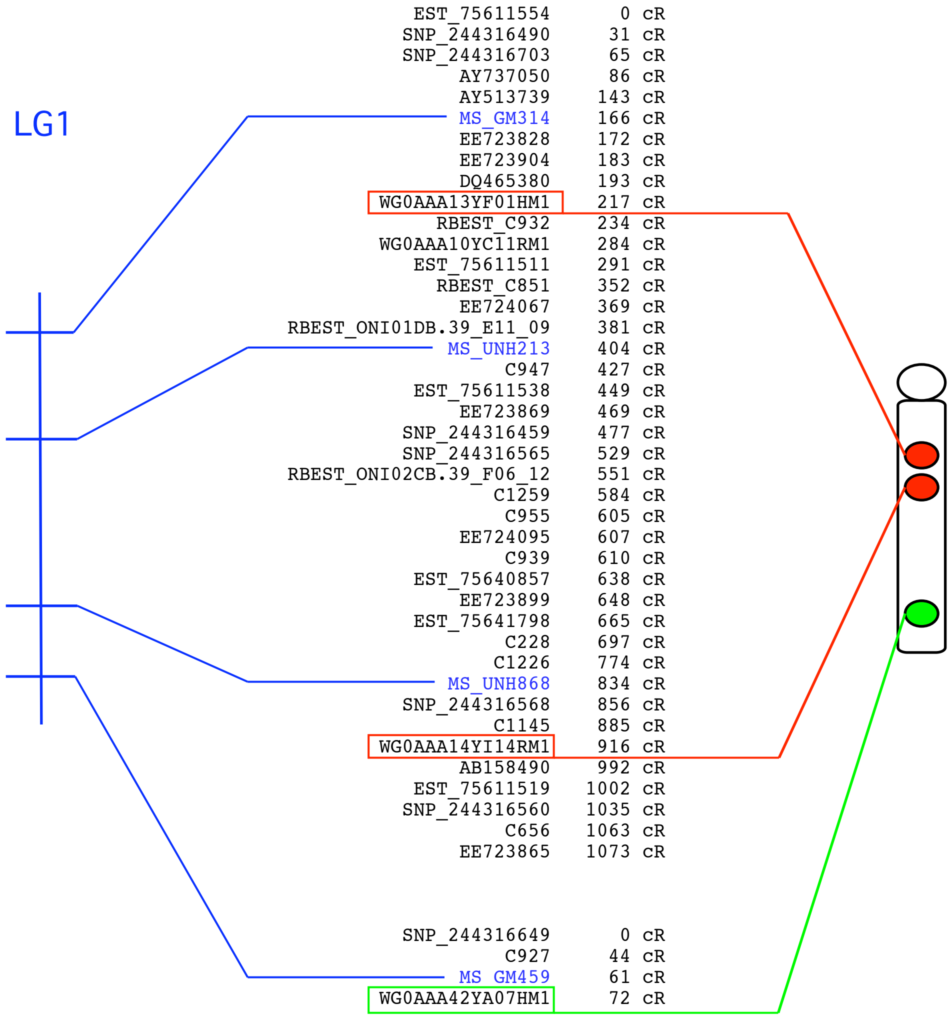

LG1
EST_75611554 0 cR
SNP_244316490 31 cR
SNP_244316703 65 cR
AY737050 86 cR
AY513739 143 cR
MS_GM314 166 cR
EE723828 172 cR
EE723904 183 cR
DQ465380 193 cR
WG0AAA13YF01HM1 217 cR
RBEST_C932 234 cR
WG0AAA10YC11RM1 284 cR
EST_75611511 291 cR
RBEST_C851 352 cR
EE724067 369 cR
RBEST_ONI01DB.39_E11_09 381 cR
MS_UNH213 404 cR
C947 427 cR
EST_75611538 449 cR
EE723869 469 cR
SNP_244316459 477 cR
SNP_244316565 529 cR
RBEST_ONI02CB.39_F06_12 551 cR
C1259 584 cR
C955 605 cR
EE724095 607 cR
C939 610 cR
EST_75640857 638 cR
EE723899 648 cR
EST_75641798 665 cR
C228 697 cR
C1226 774 cR
MS_UNH868 834 cR
SNP_244316568 856 cR
C1145 885 cR
WG0AAA14YI14RM1 916 cR
AB158490 992 cR
EST_75611519 1002 cR
SNP_244316560 1035 cR
C656 1063 cR
EE723865 1073 cR
SNP_244316649 0 cR
C927 44 cR
MS_GM459 61 cR
WG0AAA42YA07HM1 72 cR

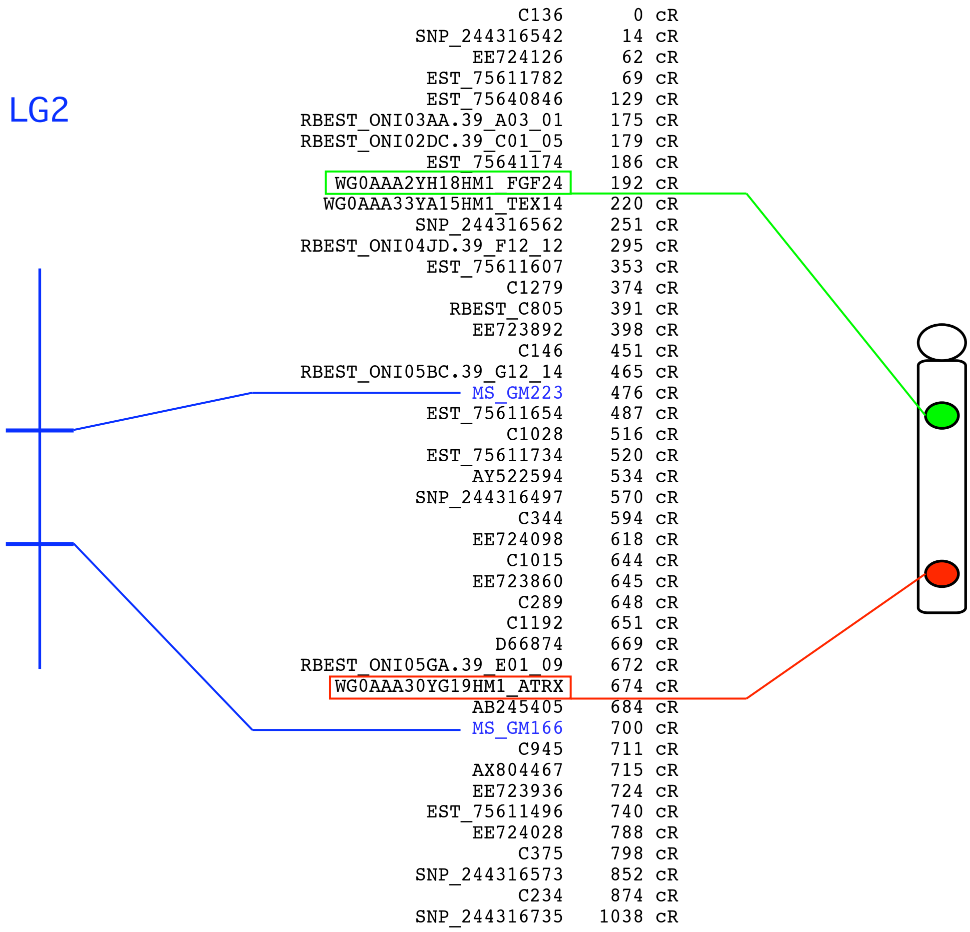
LG2
C136 0 cR
SNP_244316542 14 cR
EE724126 62 cR
EST_75611782 69 cR
EST_75640846 129 cR
RBEST_ONI03AA.39_A03_01 175 cR
RBEST_ONI02DC.39_C01_05 179 cR
EST_75641174 186 cR
WG0AAA2YH18HM1_FGF24 192 cR
WG0AAA33YA15HM1_TEX14 220 cR
SNP_244316562 251 cR
RBEST_ONI04JD.39_F12_12 295 cR
EST_75611607 353 cR
C1279 374 cR
RBEST_C805 391 cR
EE723892 398 cR
C146 451 cR
RBEST_ONI05BC.39_G12_14 465 cR
MS_GM223 476 cR
EST_75611654 487 cR
C1028 516 cR
EST_75611734 520 cR
AY522594 534 cR
SNP_244316497 570 cR
C344 594 cR
EE724098 618 cR
C1015 644 cR
EE723860 645 cR
C289 648 cR
C1192 651 cR
D66874 669 cR
RBEST_ONI05GA.39_E01_09 672 cR
WG0AAA30YG19HM1_ATRX 674 cR
AB245405 684 cR
MS_GM166 700 cR
C945 711 cR
AX804467 715 cR
EE723936 724 cR
EST_75611496 740 cR
EE724028 788 cR
C375 798 cR
SNP_244316573 852 cR
C234 874 cR
SNP_244316735 1038 cR

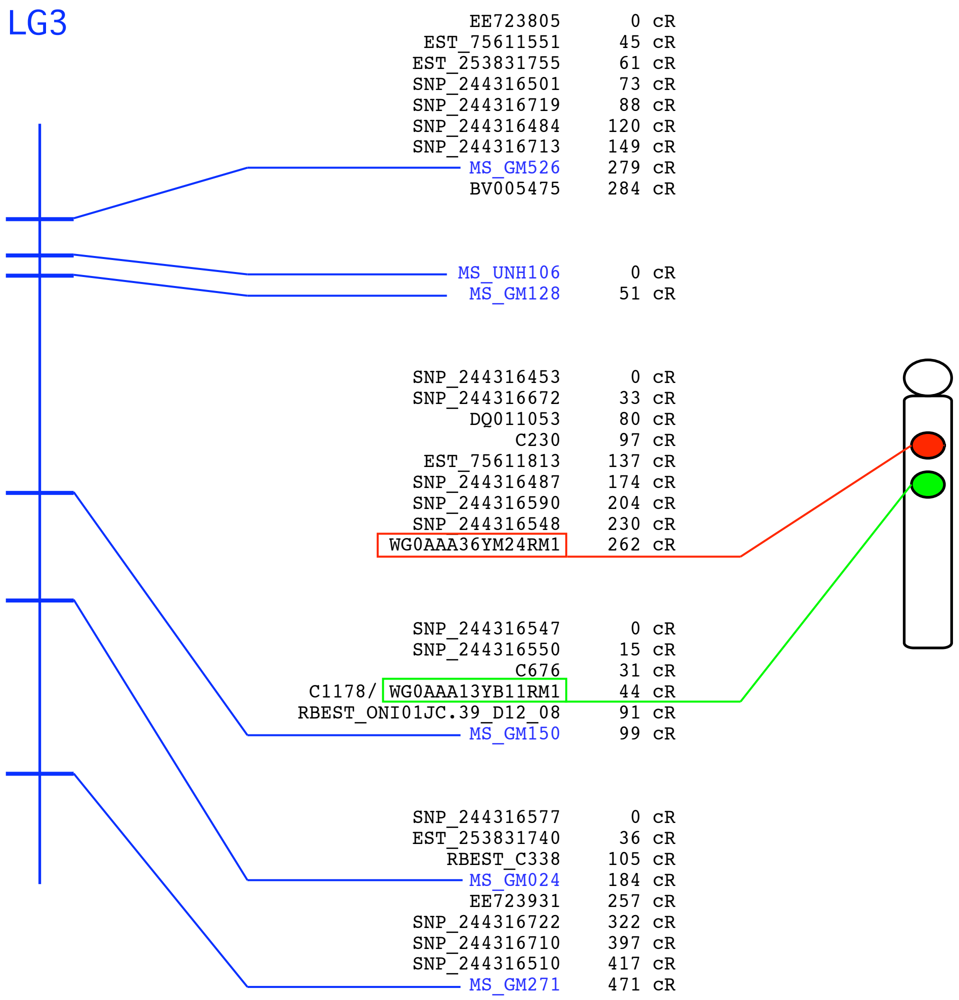
LG3
EE723805 0 cR
EST_75611551 45 cR
EST_253831755 61 cR
SNP_244316501 73 cR
SNP_244316719 88 cR
SNP_244316484 120 cR
SNP_244316713 149 cR
MS_GM526 279 cR
BV005475 284 cR
MS_UNH106 0 cR
MS_GM128 51 cR
SNP_244316453 0 cR
SNP_244316672 33 cR
DQ011053 80 cR
C230 97 cR
EST_75611813 137 cR
SNP_244316487 174 cR
SNP_244316590 204 cR
SNP_244316548 230 cR
WG0AAA36YM24RM1 262 cR
SNP_244316547 0 cR
SNP_244316550 15 cR
C676 31 cR
C1178/ WG0AAA13YB11RM1 44 cR
RBEST_ONI01JC.39_D12_08 91 cR
MS_GM150 99 cR
SNP_244316577 0 cR
EST_253831740 36 cR
RBEST_C338 105 cR
MS_GM024 184 cR
EE723931 257 cR
SNP_244316722 322 cR
SNP_244316710 397 cR
SNP_244316510 417 cR
MS_GM271 471 cR

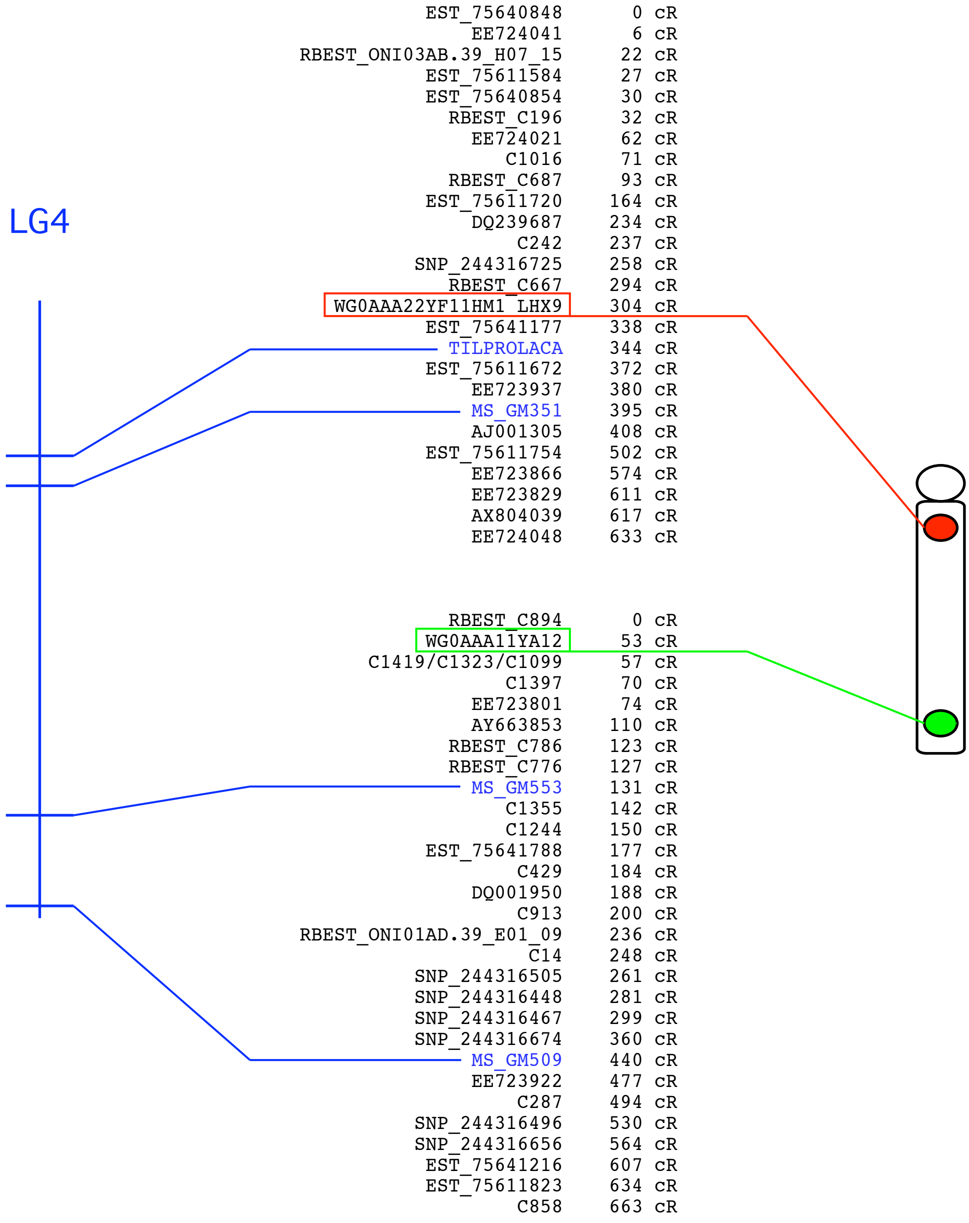
LG4
EST_75640848 0 cR
EE724041 6 cR
RBEST_ONI03AB.39_H07_15 22 cR
EST_75611584 27 cR
EST_75640854 30 cR
RBEST_C196 32 cR
EE724021 62 cR
C1016 71 cR
RBEST_C687 93 cR
EST_75611720 164 cR
DQ239687 234 cR
C242 237 cR
SNP_244316725 258 cR
RBEST_C667 294 cR
WG0AAA22YF11HM1_LHX9 304 cR
EST_75641177 338 cR
TILPROLACA 344 cR
EST_75611672 372 cR
EE723937 380 cR
MS_GM351 395 cR
AJ001305 408 cR
EST_75611754 502 cR
EE723866 574 cR
EE723829 611 cR
AX804039 617 cR
EE724048 633 cR
RBEST_C894 0 cR
WG0AAA11YA12 53 cR
C1419/C1323/C1099 57 cR
C1397 70 cR
EE723801 74 cR
AY663853 110 cR
RBEST_C786 123 cR
RBEST_C776 127 cR
MS_GM553 131 cR
C1355 142 cR
C1244 150 cR
EST_75641788 177 cR
C429 184 cR
DQ001950 188 cR
C913 200 cR
RBEST_ONI01AD.39_E01_09 236 cR
C14 248 cR
SNP_244316505 261 cR
SNP_244316448 281 cR
SNP_244316467 299 cR
SNP_244316674 360 cR
MS_GM509 440 cR
EE723922 477 cR
C287 494 cR
SNP_244316496 530 cR
SNP_244316656 564 cR
EST_75641216 607 cR
EST_75611823 634 cR
C858 663 cR

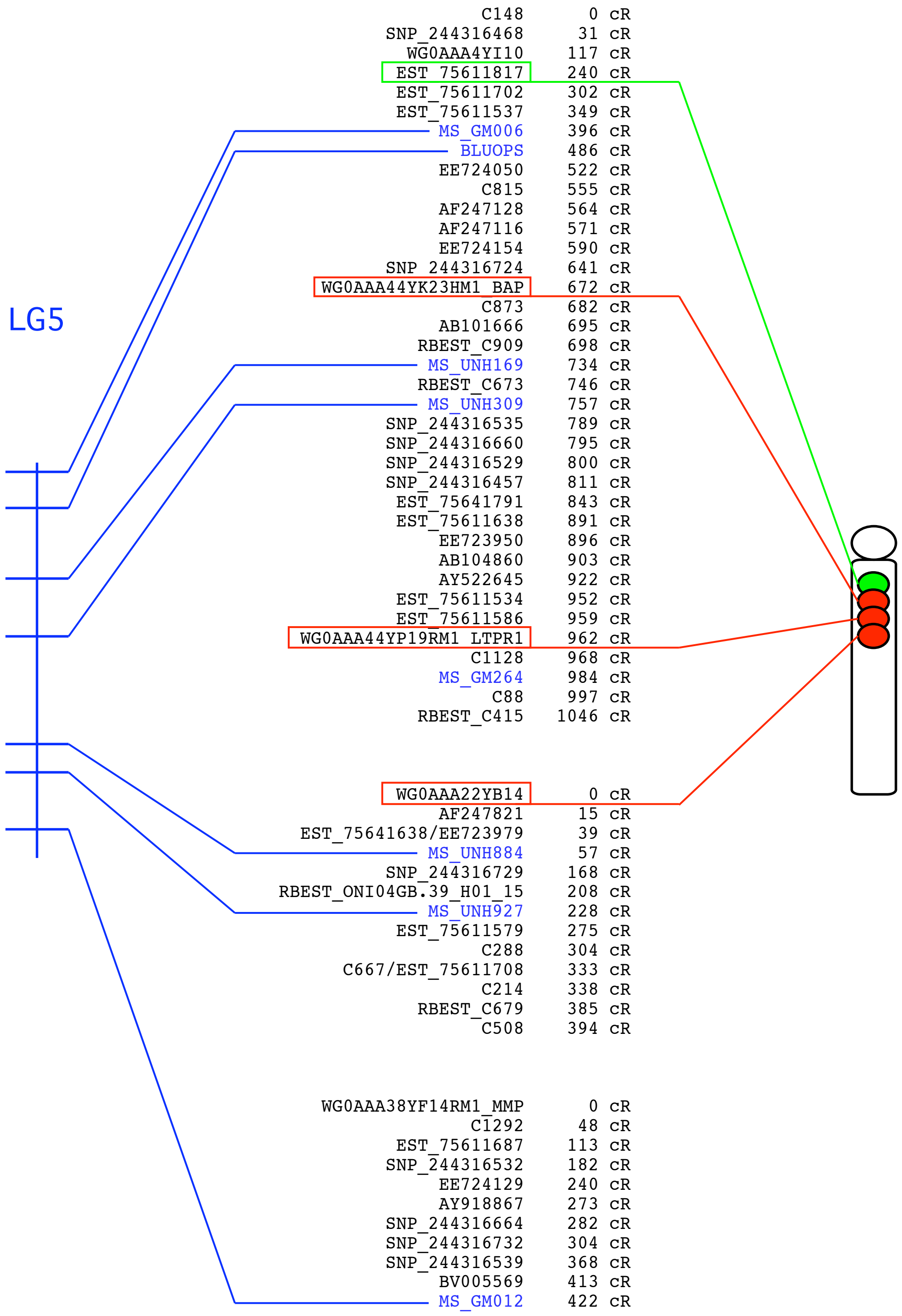

LG5
C148 0 cR
SNP_244316468 31 cR
WG0AAA4YI10 117 cR
EST_75611817 240 cR
EST_75611702 302 cR
EST_75611537 349 cR
MS_GM006 396 cR
BLUOPS 486 cR
EE724050 522 cR
C815 555 cR
AF247128 564 cR
AF247116 571 cR
EE724154 590 cR
SNP_244316724 641 cR
WG0AAA44YK23HM1_BAP 672 cR
C873 682 cR
AB101666 695 cR
RBEST_C909 698 cR
MS_UNH169 734 cR
RBEST_C673 746 cR
MS_UNH309 757 cR
SNP_244316535 789 cR
SNP_244316660 795 cR
SNP_244316529 800 cR
SNP_244316457 811 cR
EST_75641791 843 cR
EST_75611638 891 cR
EE723950 896 cR
AB104860 903 cR
AY522645 922 cR
EST_75611534 952 cR
EST_75611586 959 cR
WG0AAA44YP19RM1_LTPR1 962 cR
C1128 968 cR
MS_GM264 984 cR
C88 997 cR
RBEST_C415 1046 cR
WG0AAA22YB14 0 cR
AF247821 15 cR
EST_75641638/EE723979 39 cR
MS_UNH884 57 cR
SNP_244316729 168 cR
RBEST_ONI04GB.39_H01_15 208 cR
MS_UNH927 228 cR
EST_75611579 275 cR
C288 304 cR
C667/EST_75611708 333 cR
C214 338 cR
RBEST_C679 385 cR
C508 394 cR
WG0AAA38YF14RM1_MMP 0 cR
C1292 48 cR
EST_75611687 113 cR
SNP_244316532 182 cR
EE724129 240 cR
AY918867 273 cR
SNP_244316664 282 cR
SNP_244316732 304 cR
SNP_244316539 368 cR
BV005569 413 cR
MS_GM012 422 cR

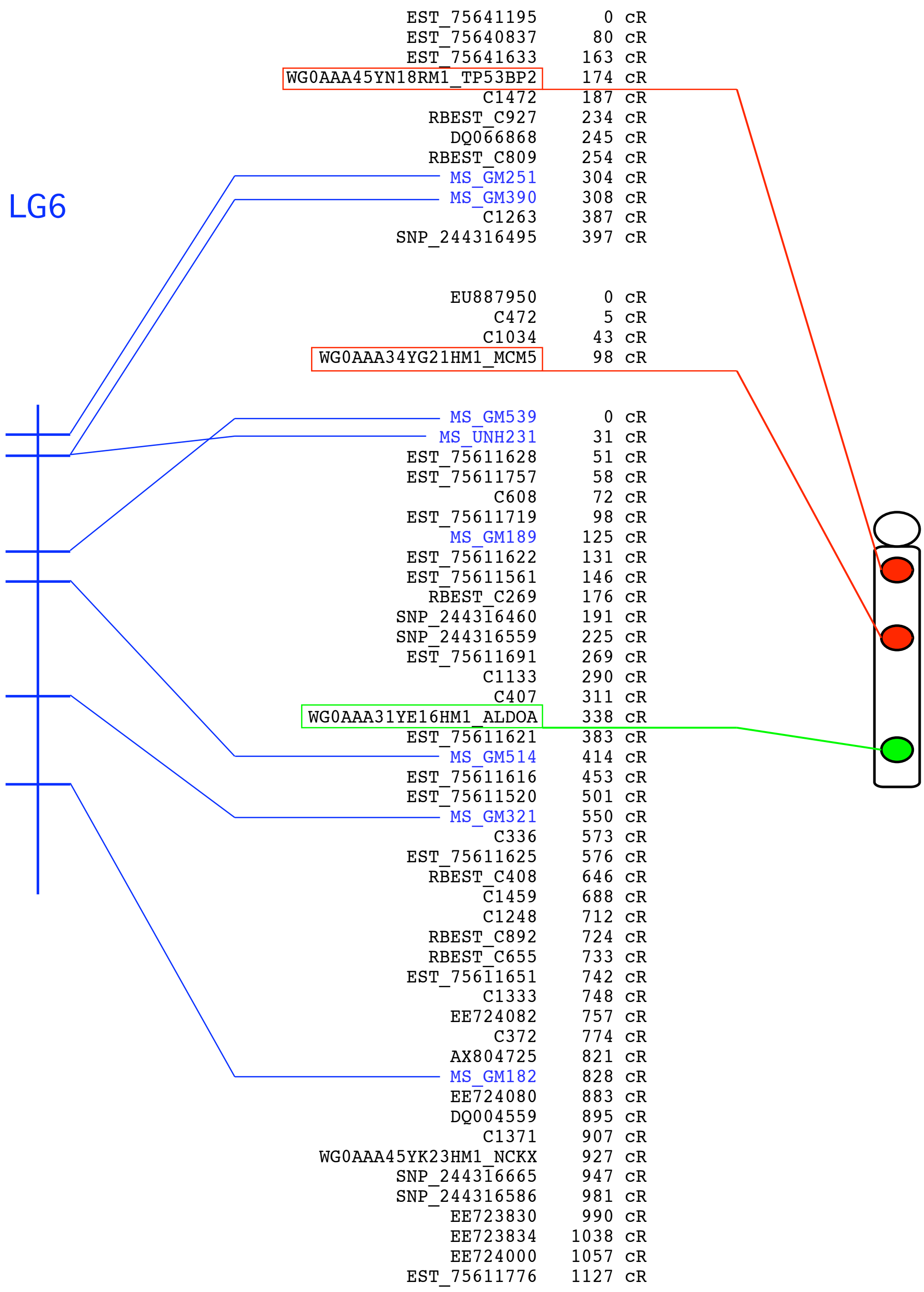

LG6
EST_75641195 0 cR
EST_75640837 80 cR
EST_75641633 163 cR
WG0AAA45YN18RM1_TP53BP2 174 cR
C1472 187 cR
RBEST_C927 234 cR
DQ066868 245 cR
RBEST_C809 254 cR
MS_GM251 304 cR
MS_GM390 308 cR
C1263 387 cR
SNP_244316495 397 cR
EU887950 0 cR
C472 5 cR
C1034 43 cR
WG0AAA34YG21HM1_MCM5 98 cR
MS_GM539 0 cR
MS_UNH231 31 cR
EST_75611628 51 cR
EST_75611757 58 cR
C608 72 cR
EST_75611719 98 cR
MS_GM189 125 cR
EST_75611622 131 cR
EST_75611561 146 cR
RBEST_C269 176 cR
SNP_244316460 191 cR
SNP_244316559 225 cR
EST_75611691 269 cR
C1133 290 cR
C407 311 cR
WG0AAA31YE16HM1_ALDOA 338 cR
EST_75611621 383 cR
MS_GM514 414 cR
EST_75611616 453 cR
EST_75611520 501 cR
MS_GM321 550 cR
C336 573 cR
EST_75611625 576 cR
RBEST_C408 646 cR
C1459 688 cR
C1248 712 cR
RBEST_C892 724 cR
RBEST_C655 733 cR
EST_75611651 742 cR
C1333 748 cR
EE724082 757 cR
C372 774 cR
AX804725 821 cR
MS_GM182 828 cR
EE724080 883 cR
DQ004559 895 cR
C1371 907 cR
WG0AAA45YK23HM1_NCKX 927 cR
SNP_244316665 947 cR
SNP_244316586 981 cR
EE723830 990 cR
EE723834 1038 cR
EE724000 1057 cR
EST_75611776 1127 cR

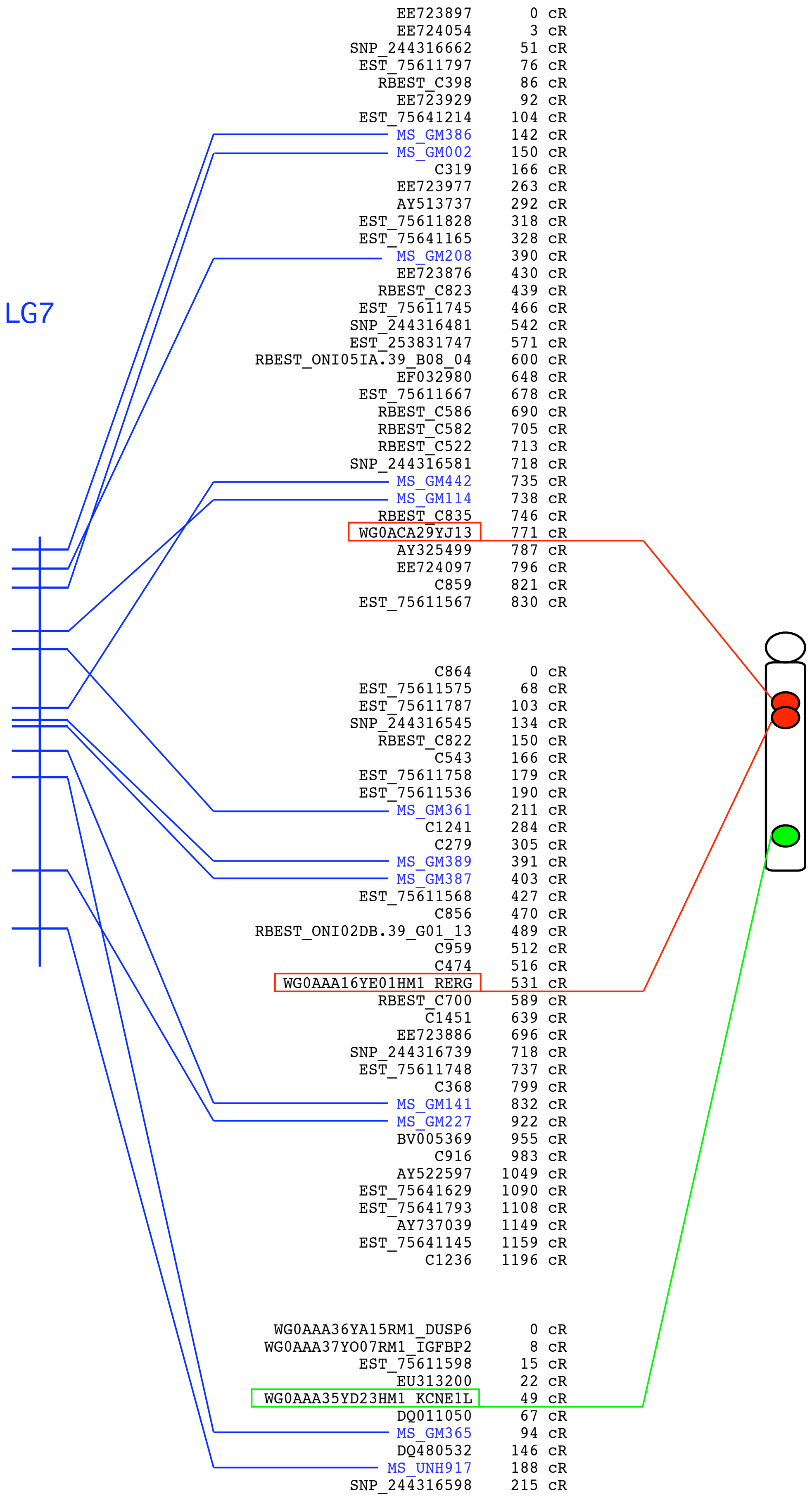
LG7
EE723897 0 cR
EE724054 3 cR
SNP_244316662 51 cR
EST_75611797 76 cR
RBEST_C398 86 cR
EE723929 92 cR
EST_75641214 104 cR
MS_GM386 142 cR
MS_GM002 150 cR
C319 166 cR
EE723977 263 cR
AY513737 292 cR
EST_75611828 318 cR
EST_75641165 328 cR
MS_GM208 390 cR
EE723876 430 cR
RBEST_C823 439 cR
EST_75611745 466 cR
SNP_244316481 542 cR
EST_253831747 571 cR
RBEST_ONI05IA.39_B08_04 600 cR
EF032980 648 cR
EST_75611667 678 cR
RBEST_C586 690 cR
RBEST_C582 705 cR
RBEST_C522 713 cR
SNP_244316581 718 cR
MS_GM442 735 cR
MS_GM114 738 cR
RBEST_C835 746 cR
WG0ACA29YJ13 771 cR
AY325499 787 cR
EE724097 796 cR
C859 821 cR
EST_75611567 830 cR
C864 0 cR
EST_75611575 68 cR
EST_75611787 103 cR
SNP_244316545 134 cR
RBEST_C822 150 cR
C543 166 cR
EST_75611758 179 cR
EST_75611536 190 cR
MS_GM361 211 cR
C1241 284 cR
C279 305 cR
MS_GM389 391 cR
MS_GM387 403 cR
EST_75611568 427 cR
C856 470 cR
RBEST_ONI02DB.39_G01_13 489 cR
C959 512 cR
C474 516 cR
WG0AAA16YE01HM1_RERG 531 cR
RBEST_C700 589 cR
C1451 639 cR
EE723886 696 cR
SNP_244316739 718 cR
EST_75611748 737 cR
C368 799 cR
MS_GM141 832 cR
MS_GM227 922 cR
BV005369 955 cR
C916 983 cR
AY522597 1049 cR
EST_75641629 1090 cR
EST_75641793 1108 cR
AY737039 1149 cR
EST_75641145 1159 cR
C1236 1196 cR
WG0AAA36YA15RM1_DUSP6 0 cR
WG0AAA37YO07RM1_IGFBP2 8 cR
EST_75611598 15 cR
EU313200 22 cR
WG0AAA35YD23HM1_KCNE1L 49 cR
DQ011050 67 cR
MS_GM365 94 cR
DQ480532 146 cR
MS_UNH917 188 cR
SNP_244316598 215 cR

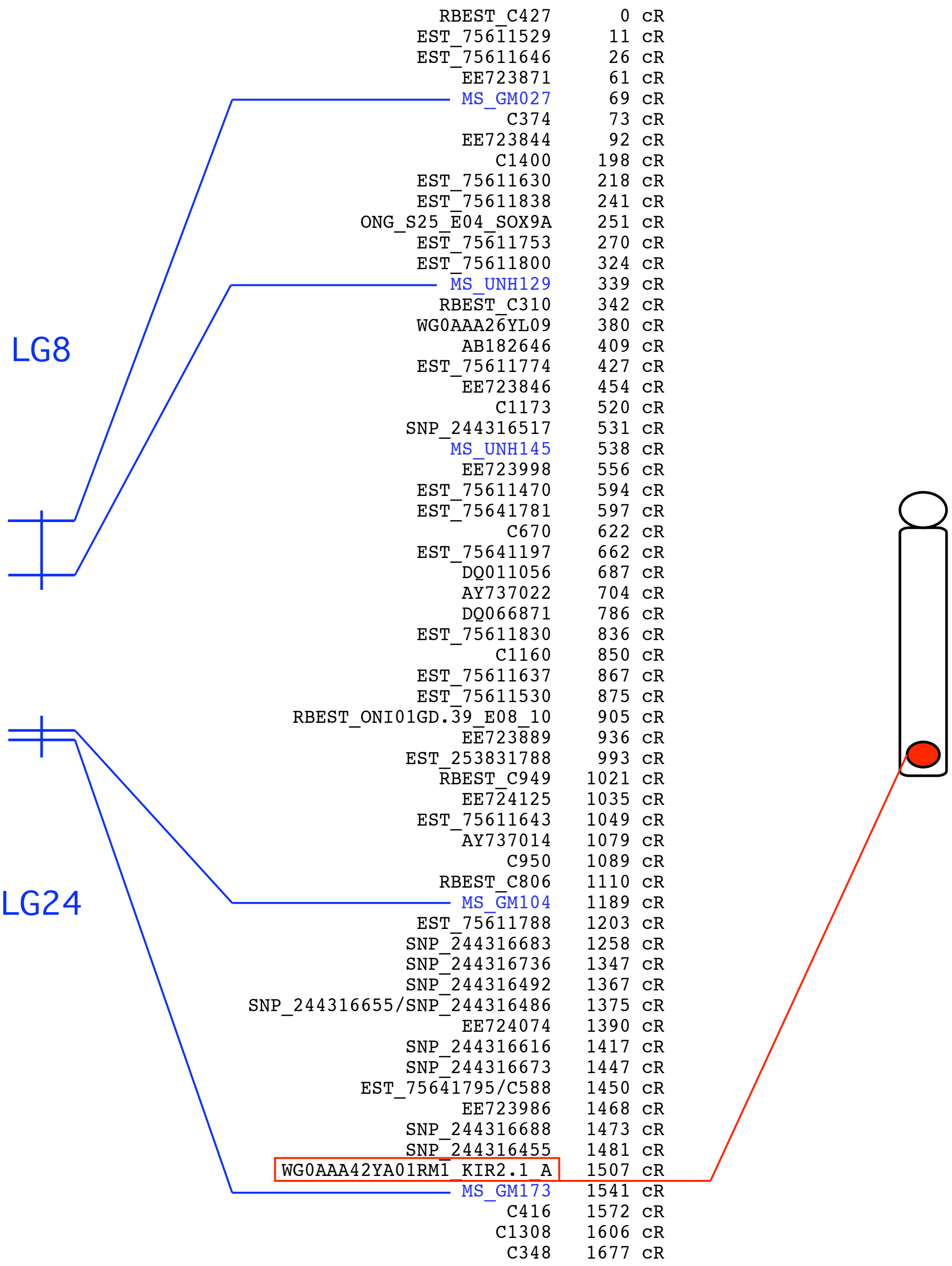
LG8
LG24
RBEST_C427 0 cR
EST_75611529 11 cR
EST_75611646 26 cR
EE723871 61 cR
MS_GM027 69 cR
C374 73 cR
EE723844 92 cR
C1400 198 cR
EST_75611630 218 cR
EST_75611838 241 cR
ONG_S25_E04_SOX9A 251 cR
EST_75611753 270 cR
EST_75611800 324 cR
MS_UNH129 339 cR
RBEST_C310 342 cR
WG0AAA26YL09 380 cR
AB182646 409 cR
EST_75611774 427 cR
EE723846 454 cR
C1173 520 cR
SNP_244316517 531 cR
MS_UNH145 538 cR
EE723998 556 cR
EST_75611470 594 cR
EST_75641781 597 cR
C670 622 cR
EST_75641197 662 cR
DQ011056 687 cR
AY737022 704 cR
DQ066871 786 cR
EST_75611830 836 cR
C1160 850 cR
EST_75611637 867 cR
EST_75611530 875 cR
RBEST_ONI01GD.39_E08_10 905 cR
EE723889 936 cR
EST_253831788 993 cR
RBEST_C949 1021 cR
EE724125 1035 cR
EST_75611643 1049 cR
AY737014 1079 cR
C950 1089 cR
RBEST_C806 1110 cR
MS_GM104 1189 cR
EST_75611788 1203 cR
SNP_244316683 1258 cR
SNP_244316736 1347 cR
SNP_244316492 1367 cR
SNP_244316655/SNP_244316486 1375 cR
EE724074 1390 cR
SNP_244316616 1417 cR
SNP_244316673 1447 cR
EST_75641795/C588 1450 cR
EE723986 1468 cR
SNP_244316688 1473 cR
SNP_244316455 1481 cR
WG0AAA42YA01RM1_KIR2.1_A 1507 cR
MS_GM173 1541 cR
C416 1572 cR
C1308 1606 cR
C348 1677 cR

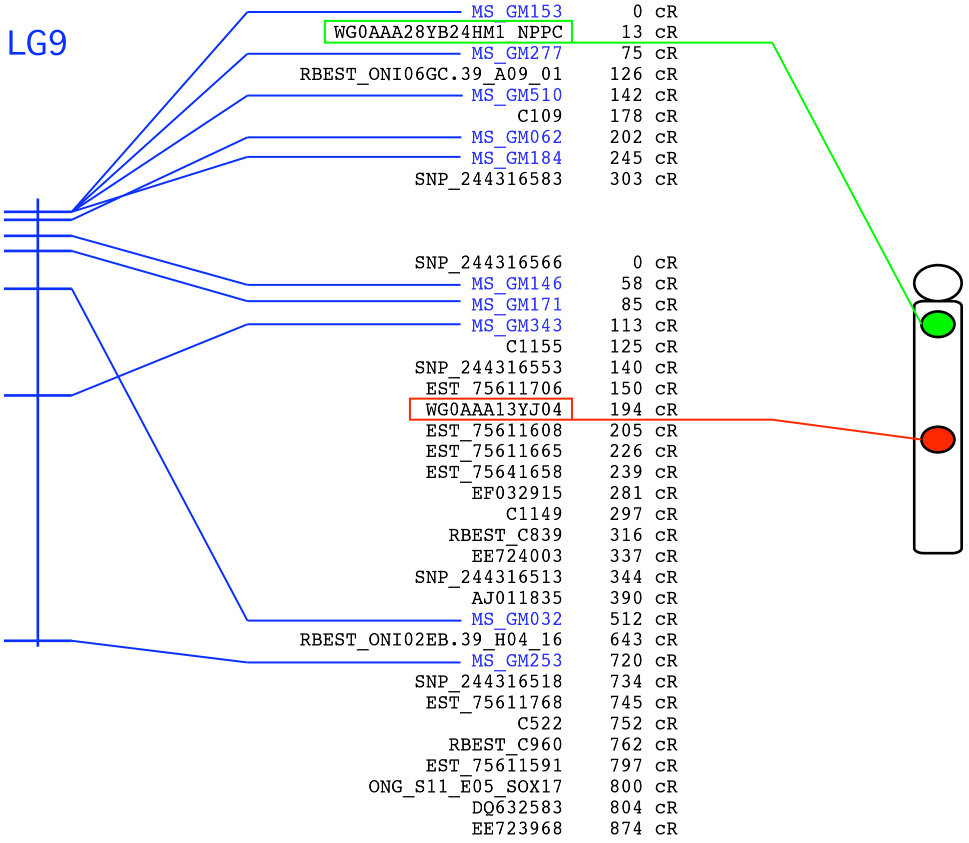

LG9
MS_GM153 0 cR
WG0AAA28YB24HM1_NPPC 13 cR
MS_GM277 75 cR
RBEST_ONI06GC.39_A09_01 126 cR
MS_GM510 142 cR
C109 178 cR
MS_GM062 202 cR
MS_GM184 245 cR
SNP_244316583 303 cR
SNP_244316566 0 cR
MS_GM146 58 cR
MS_GM171 85 cR
MS_GM343 113 cR
C1155 125 cR
SNP_244316553 140 cR
EST_75611706 150 cR
WG0AAA13YJ04 194 cR
EST_75611608 205 cR
EST_75611665 226 cR
EST_75641658 239 cR
EF032915 281 cR
C1149 297 cR
RBEST_C839 316 cR
EE724003 337 cR
SNP_244316513 344 cR
AJ011835 390 cR
MS_GM032 512 cR
RBEST_ONI02EB.39_H04_16 643 cR
MS_GM253 720 cR
SNP_244316518 734 cR
EST_75611768 745 cR
C522 752 cR
RBEST_C960 762 cR
EST_75611591 797 cR
ONG_S11_E05_SOX17 800 cR
DQ632583 804 cR
EE723968 874 cR

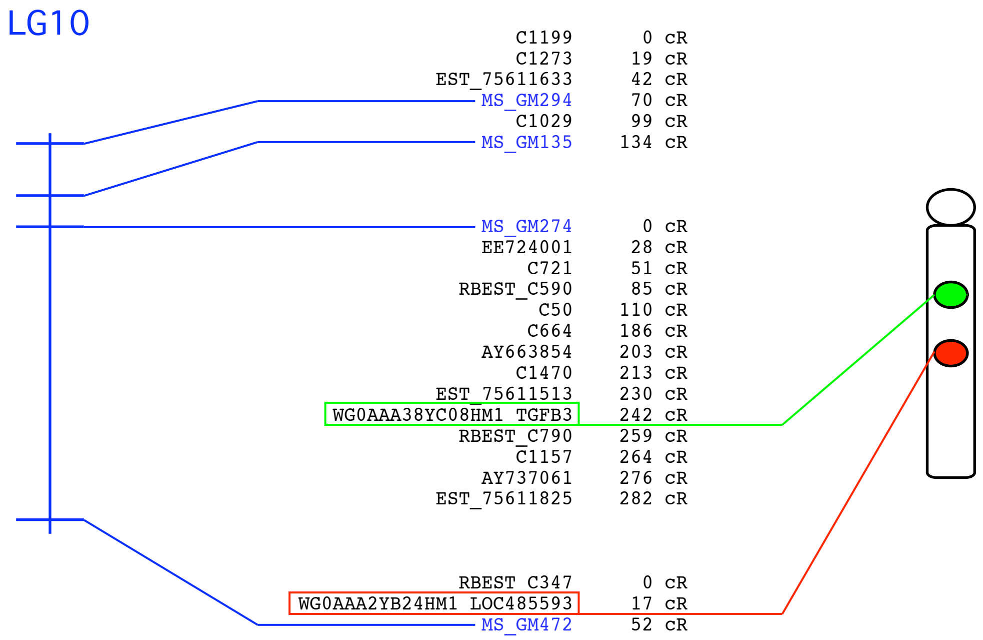
LG10
C1199 0 cR
C1273 19 cR
EST_75611633 42 cR
MS_GM294 70 cR
C1029 99 cR
MS_GM135 134 cR
MS_GM274 0 cR
EE724001 28 cR
C721 51 cR
RBEST_C590 85 cR
C50 110 cR
C664 186 cR
AY663854 203 cR
C1470 213 cR
EST_75611513 230 cR
WG0AAA38YC08HM1_TGFB3 242 cR
RBEST_C790 259 cR
C1157 264 cR
AY737061 276 cR
EST_75611825 282 cR
RBEST_C347 0 cR
WG0AAA2YB24HM1_LOC485593 17 cR
MS_GM472 52 cR

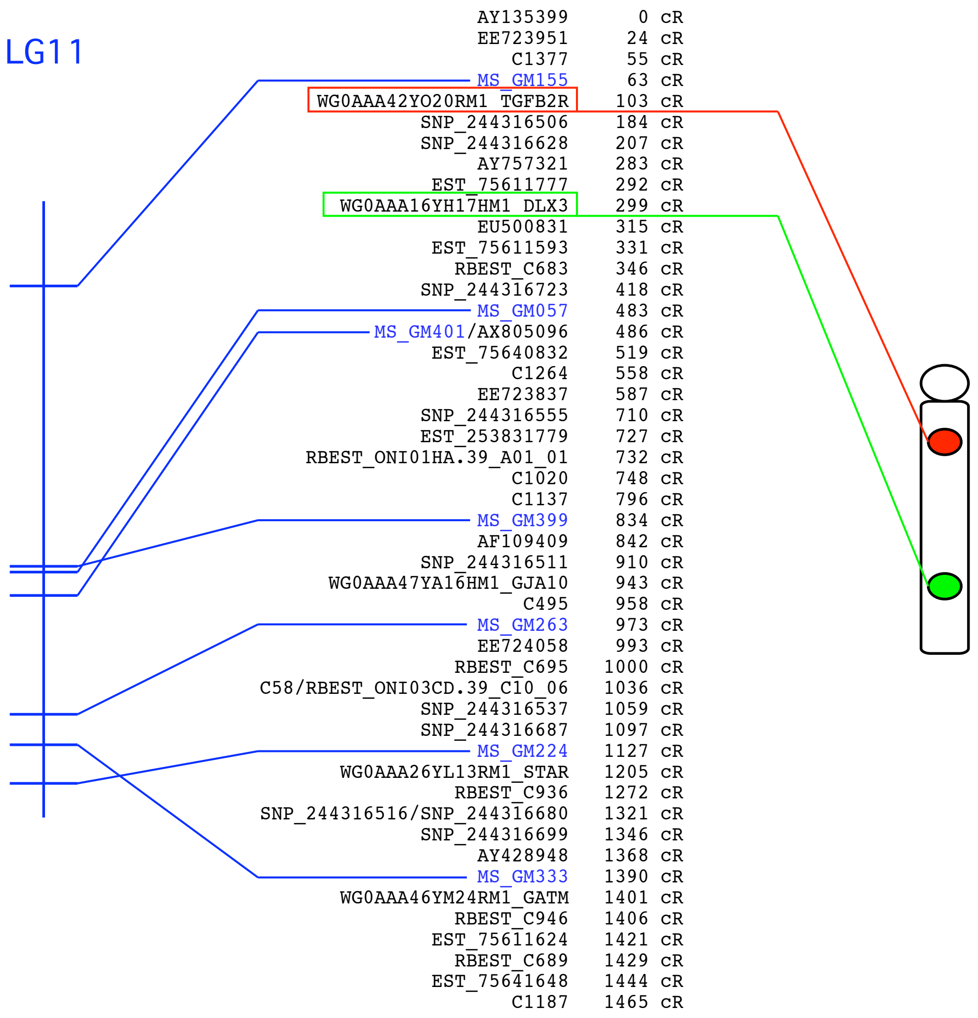
LG11
AY135399 0 cR
EE723951 24 cR
C1377 55 cR
MS_GM155 63 cR
WG0AAA42YO20RM1_TGFB2R 103 cR
SNP_244316506 184 cR
SNP_244316628 207 cR
AY757321 283 cR
EST_75611777 292 cR
WG0AAA16YH17HM1_DLX3 299 cR
EU500831 315 cR
EST_75611593 331 cR
RBEST_C683 346 cR
SNP_244316723 418 cR
MS_GM057 483 cR
MS_GM401/AX805096 486 cR
EST_75640832 519 cR
C1264 558 cR
EE723837 587 cR
SNP_244316555 710 cR
EST_253831779 727 cR
RBEST_ONI01HA.39_A01_01 732 cR
C1020 748 cR
C1137 796 cR
MS_GM399 834 cR
AF109409 842 cR
SNP_244316511 910 cR
WG0AAA47YA16HM1_GJA10 943 cR
C495 958 cR
MS_GM263 973 cR
EE724058 993 cR
RBEST_C695 1000 cR
C58/RBEST_ONI03CD.39_C10_06 1036 cR
SNP_244316537 1059 cR
SNP_244316687 1097 cR
MS_GM224 1127 cR
WG0AAA26YL13RM1_STAR 1205 cR
RBEST_C936 1272 cR
SNP_244316516/SNP_244316680 1321 cR
SNP_244316699 1346 cR
AY428948 1368 cR
MS_GM333 1390 cR
WG0AAA46YM24RM1_GATM 1401 cR
RBEST_C946 1406 cR
EST_75611624 1421 cR
RBEST_C689 1429 cR
EST_75641648 1444 cR
C1187 1465 cR

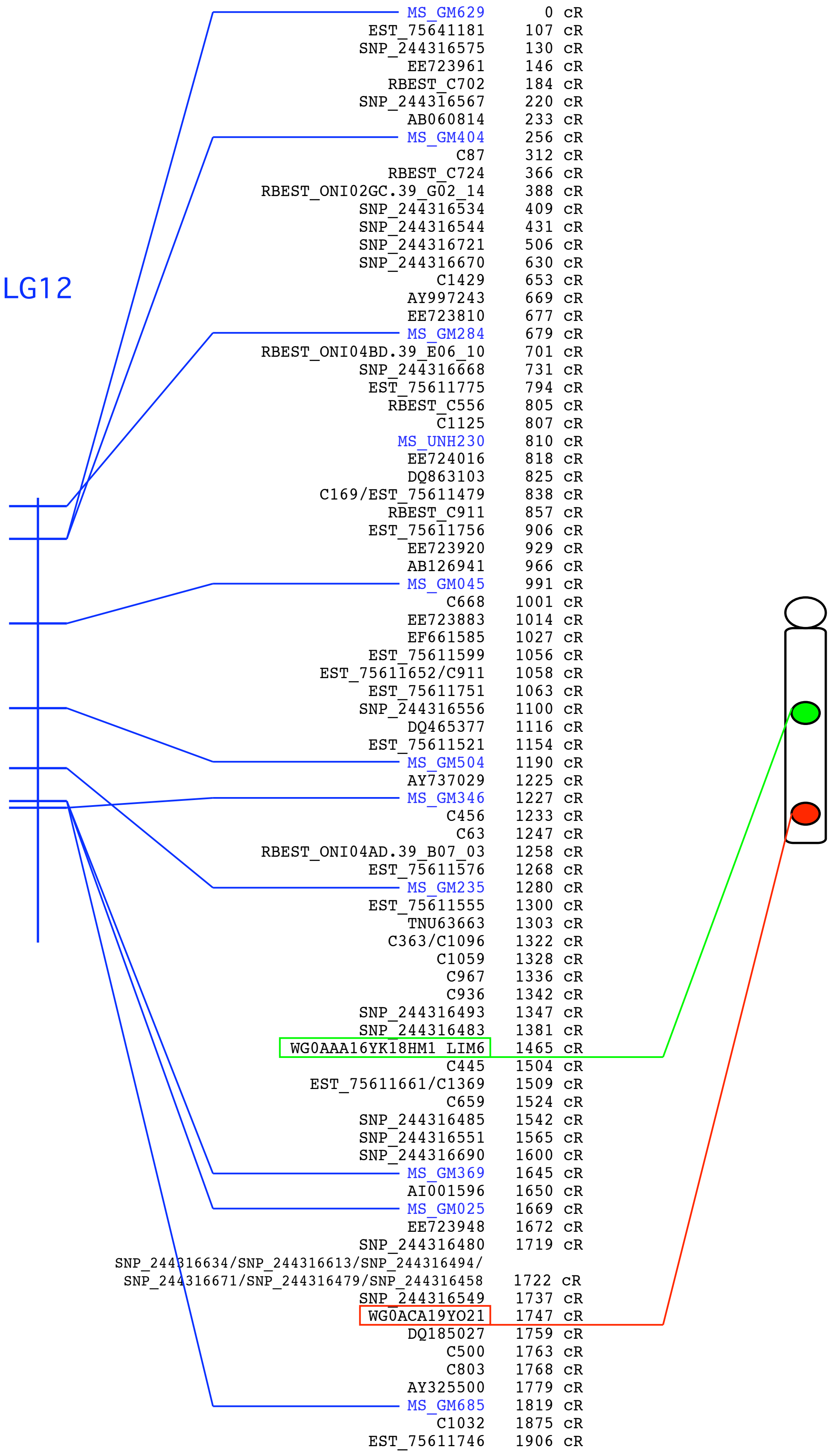
LG12
MS_GM629 0 cR
EST_75641181 107 cR
SNP_244316575 130 cR
EE723961 146 cR
RBEST_C702 184 cR
SNP_244316567 220 cR
AB060814 233 cR
MS_GM404 256 cR
C87 312 cR
RBEST_C724 366 cR
RBEST_ONI02GC.39_G02_14 388 cR
SNP_244316534 409 cR
SNP_244316544 431 cR
SNP_244316721 506 cR
SNP_244316670 630 cR
C1429 653 cR
AY997243 669 cR
EE723810 677 cR
MS_GM284 679 cR
RBEST_ONI04BD.39_E06_10 701 cR
SNP_244316668 731 cR
EST_75611775 794 cR
RBEST_C556 805 cR
C1125 807 cR
MS_UNH230 810 cR
EE724016 818 cR
DQ863103 825 cR
C169/EST_75611479 838 cR
RBEST_C911 857 cR
EST_75611756 906 cR
EE723920 929 cR
AB126941 966 cR
MS_GM045 991 cR
C668 1001 cR
EE723883 1014 cR
EF661585 1027 cR
EST_75611599 1056 cR
EST_75611652/C911 1058 cR
EST_75611751 1063 cR
SNP_244316556 1100 cR
DQ465377 1116 cR
EST_75611521 1154 cR
MS_GM504 1190 cR
AY737029 1225 cR
MS_GM346 1227 cR
C456 1233 cR
C63 1247 cR
RBEST_ONI04AD.39_B07_03 1258 cR
EST_75611576 1268 cR
MS_GM235 1280 cR
EST_75611555 1300 cR
TNU63663 1303 cR
C363/C1096 1322 cR
C1059 1328 cR
C967 1336 cR
C936 1342 cR
SNP_244316493 1347 cR
SNP_244316483 1381 cR
WG0AAA16YK18HM1_LIM6 1465 cR
C445 1504 cR
EST_75611661/C1369 1509 cR
C659 1524 cR
SNP_244316485 1542 cR
SNP_244316551 1565 cR
SNP_244316690 1600 cR
MS_GM369 1645 cR
AI001596 1650 cR
MS_GM025 1669 cR
EE723948 1672 cR
SNP_244316480 1719 cR
SNP_244316634/SNP_244316613/SNP_244316494/
SNP_244316671/SNP_244316479/SNP_244316458 1722 cR
SNP_244316549 1737 cR
WG0ACA19YO21 1747 cR
DQ185027 1759 cR
C500 1763 cR
C803 1768 cR
AY325500 1779 cR
MS_GM685 1819 cR
C1032 1875 cR
EST_75611746 1906 cR

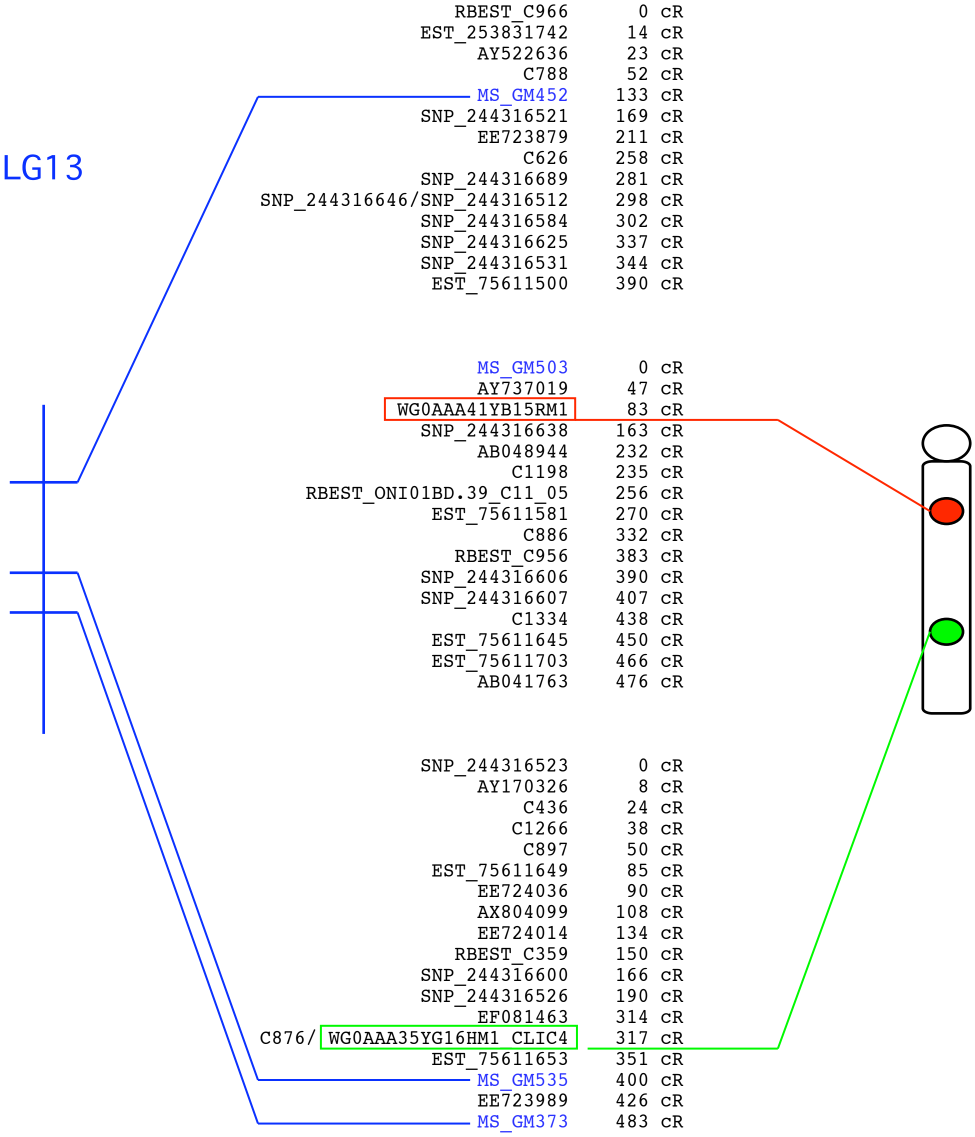
LG13
RBEST_C966 0 cR
EST_253831742 14 cR
AY522636 23 cR
C788 52 cR
MS_GM452 133 cR
SNP_244316521 169 cR
EE723879 211 cR
C626 258 cR
SNP_244316689 281 cR
SNP_244316646/SNP_244316512 298 cR
SNP_244316584 302 cR
SNP_244316625 337 cR
SNP_244316531 344 cR
EST_75611500 390 cR
MS_GM503 0 cR
AY737019 47 cR
WG0AAA41YB15RM1 83 cR
SNP_244316638 163 cR
AB048944 232 cR
C1198 235 cR
RBEST_ONI01BD.39_C11_05 256 cR
EST_75611581 270 cR
C886 332 cR
RBEST_C956 383 cR
SNP_244316606 390 cR
SNP_244316607 407 cR
C1334 438 cR
EST_75611645 450 cR
EST_75611703 466 cR
AB041763 476 cR
SNP_244316523 0 cR
AY170326 8 cR
C436 24 cR
C1266 38 cR
C897 50 cR
EST_75611649 85 cR
EE724036 90 cR
AX804099 108 cR
EE724014 134 cR
RBEST_C359 150 cR
SNP_244316600 166 cR
SNP_244316526 190 cR
EF081463 314 cR
C876/WG0AAA35YG16HM1_CLIC4 317 cR
EST_75611653 351 cR
MS_GM535 400 cR
EE723989 426 cR
MS_GM373 483 cR

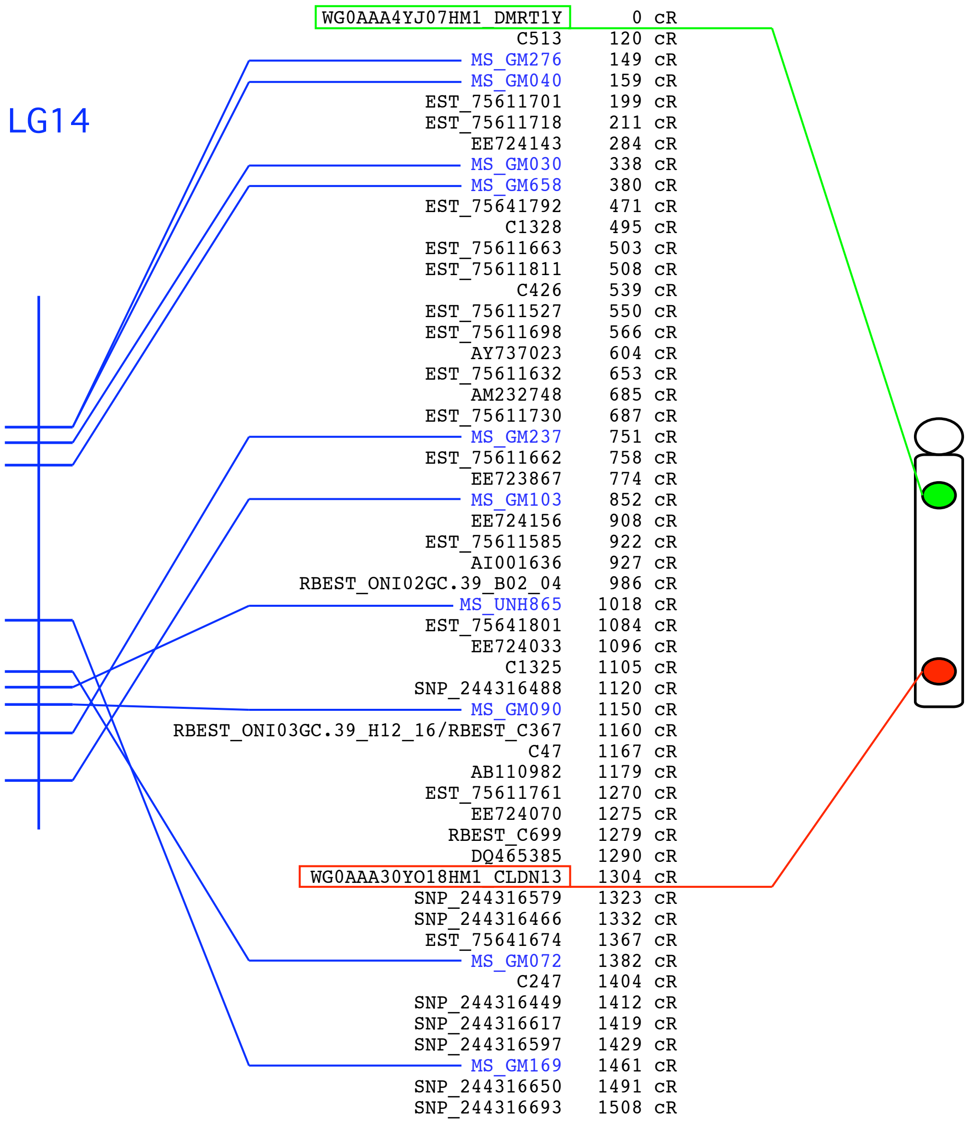
LG14
WG0AAA4YJ07HM1_DMRT1Y 0 cR
C513 120 cR
MS_GM276 149 cR
MS_GM040 159 cR
EST_75611701 199 cR
EST_75611718 211 cR
EE724143 284 cR
MS_GM030 338 cR
MS_GM658 380 cR
EST_75641792 471 cR
C1328 495 cR
EST_75611663 503 cR
EST_75611811 508 cR
C426 539 cR
EST_75611527 550 cR
EST_75611698 566 cR
AY737023 604 cR
EST_75611632 653 cR
AM232748 685 cR
EST_75611730 687 cR
MS_GM237 751 cR
EST_75611662 758 cR
EE723867 774 cR
MS_GM103 852 cR
EE724156 908 cR
EST_75611585 922 cR
AI001636 927 cR
RBEST_ONI02GC.39_B02_04 986 cR
MS_UNH865 1018 cR
EST_75641801 1084 cR
EE724033 1096 cR
C1325 1105 cR
SNP_244316488 1120 cR
MS_GM090 1150 cR
RBEST_ONI03GC.39_H12_16/RBEST_C367 1160 cR
C47 1167 cR
AB110982 1179 cR
EST_75611761 1270 cR
EE724070 1275 cR
RBEST_C699 1279 cR
DQ465385 1290 cR
WG0AAA30YO18HM1_CLDN13 1304 cR
SNP_244316579 1323 cR
SNP_244316466 1332 cR
EST_75641674 1367 cR
MS_GM072 1382 cR
C247 1404 cR
SNP_244316449 1412 cR
SNP_244316617 1419 cR
SNP_244316597 1429 cR
MS_GM169 1461 cR
SNP_244316650 1491 cR
SNP_244316693 1508 cR

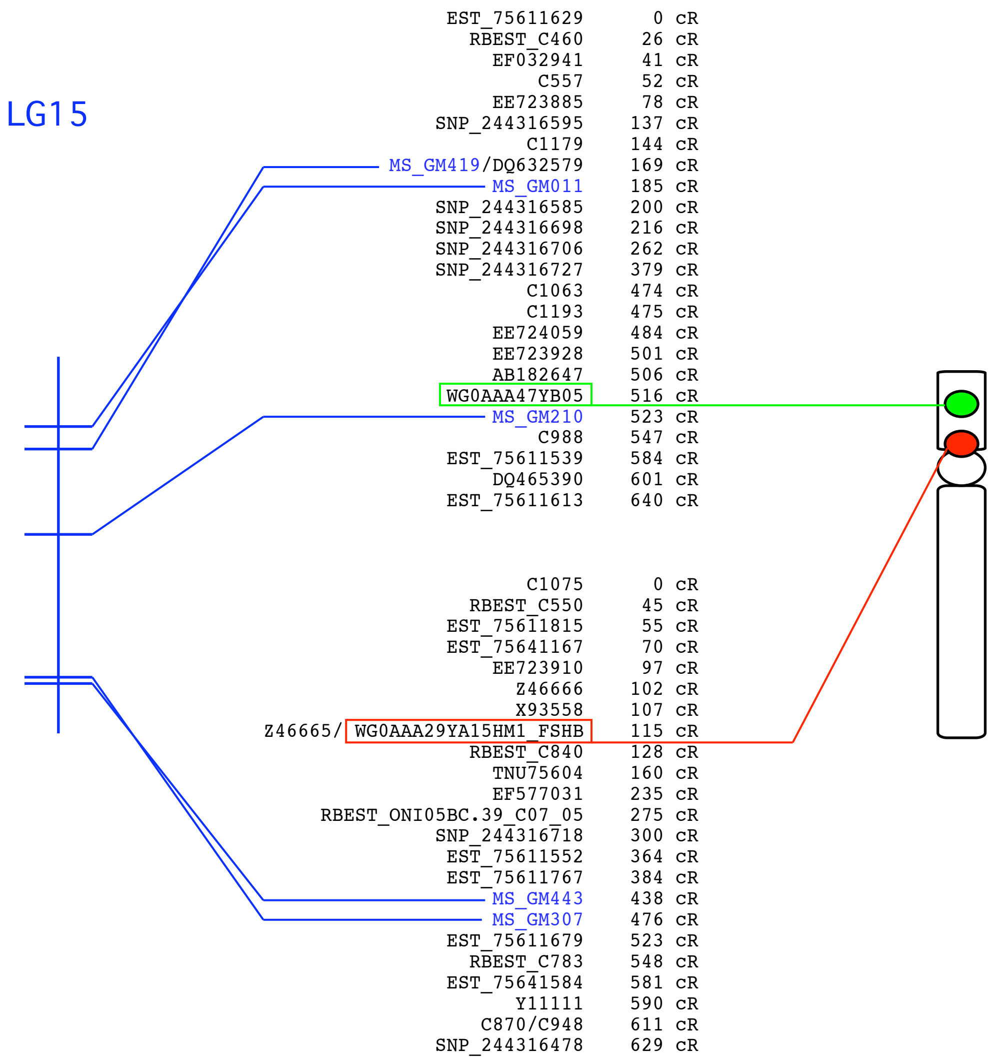

LG15
EST_75611629 0 cR
RBEST_C460 26 cR
EF032941 41 cR
C557 52 cR
EE723885 78 cR
SNP_244316595 137 cR
C1179 144 cR
MS_GM419/DQ632579 169 cR
MS_GM011 185 cR
SNP_244316585 200 cR
SNP_244316698 216 cR
SNP_244316706 262 cR
SNP_244316727 379 cR
C1063 474 cR
C1193 475 cR
EE724059 484 cR
EE723928 501 cR
AB182647 506 cR
WG0AAA47YB05 516 cR
MS_GM210 523 cR
C988 547 cR
EST_75611539 584 cR
DQ465390 601 cR
EST_75611613 640 cR
C1075 0 cR
RBEST_C550 45 cR
EST_75611815 55 cR
EST_75641167 70 cR
EE723910 97 cR
Z46666 102 cR
X93558 107 cR
Z46665/ WG0AAA29YA15HM1_FSHB 115 cR
RBEST_C840 128 cR
TNU75604 160 cR
EF577031 235 cR
RBEST_ONI05BC.39_C07_05 275 cR
SNP_244316718 300 cR
EST_75611552 364 cR
EST_75611767 384 cR
MS_GM443 438 cR
MS_GM307 476 cR
EST_75611679 523 cR
RBEST_C783 548 cR
EST_75641584 581 cR
Y11111 590 cR
C870/C948 611 cR
SNP_244316478 629 cR

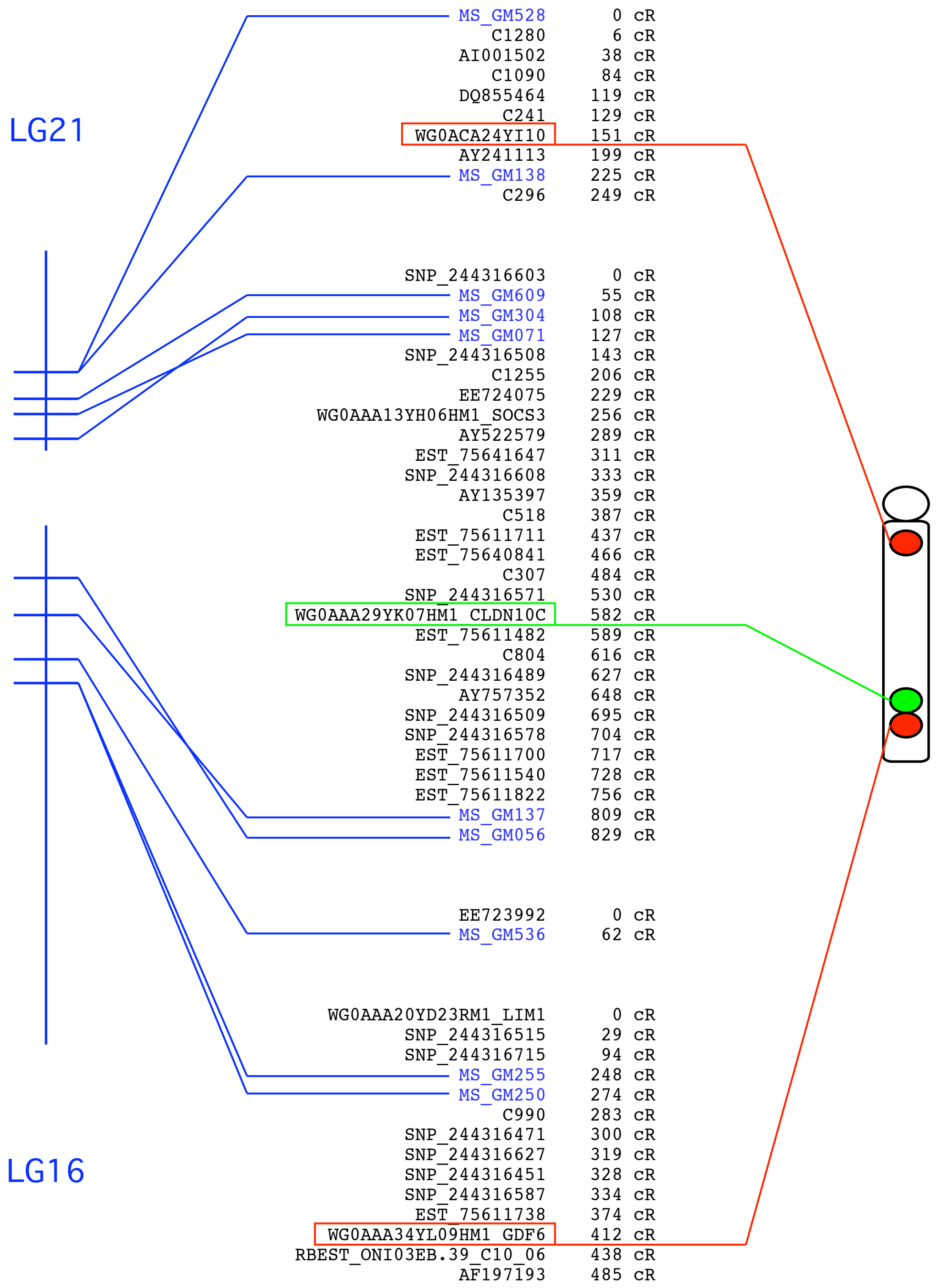
LG21
MS_GM528 0 cR
C1280 6 cR
AI001502 38 cR
C1090 84 cR
DQ855464 119 cR
C241 129 cR
WG0ACA24YI10 151 cR
AY241113 199 cR
MS_GM138 225 cR
C296 249 cR
SNP_244316603 0 cR
MS_GM609 55 cR
MS_GM304 108 cR
MS_GM071 127 cR
SNP_244316508 143 cR
C1255 206 cR
EE724075 229 cR
WG0AAA13YH06HM1_SOCS3 256 cR
AY522579 289 cR
EST_75641647 311 cR
SNP_244316608 333 cR
AY135397 359 cR
C518 387 cR
EST_75611711 437 cR
EST_75640841 466 cR
C307 484 cR
SNP_244316571 530 cR
WG0AAA29YK07HM1_CLDN10C 582 cR
EST_75611482 589 cR
C804 616 cR
SNP_244316489 627 cR
AY757352 648 cR
SNP_244316509 695 cR
SNP_244316578 704 cR
EST_75611700 717 cR
EST_75611540 728 cR
EST_75611822 756 cR
MS_GM137 809 cR
MS_GM056 829 cR
EE723992 0 cR
MS_GM536 62 cR
WG0AAA20YD23RM1_LIM1 0 cR
SNP_244316515 29 cR
SNP_244316715 94 cR
MS_GM255 248 cR
MS_GM250 274 cR
C990 283 cR
SNP_244316471 300 cR
SNP_244316627 319 cR
SNP_244316451 328 cR
SNP_244316587 334 cR
EST_75611738 374 cR
WG0AAA34YL09HM1_GDF6 412 cR
RBEST_ONI03EB.39_C10_06 438 cR
AF197193 485 cR
LG16

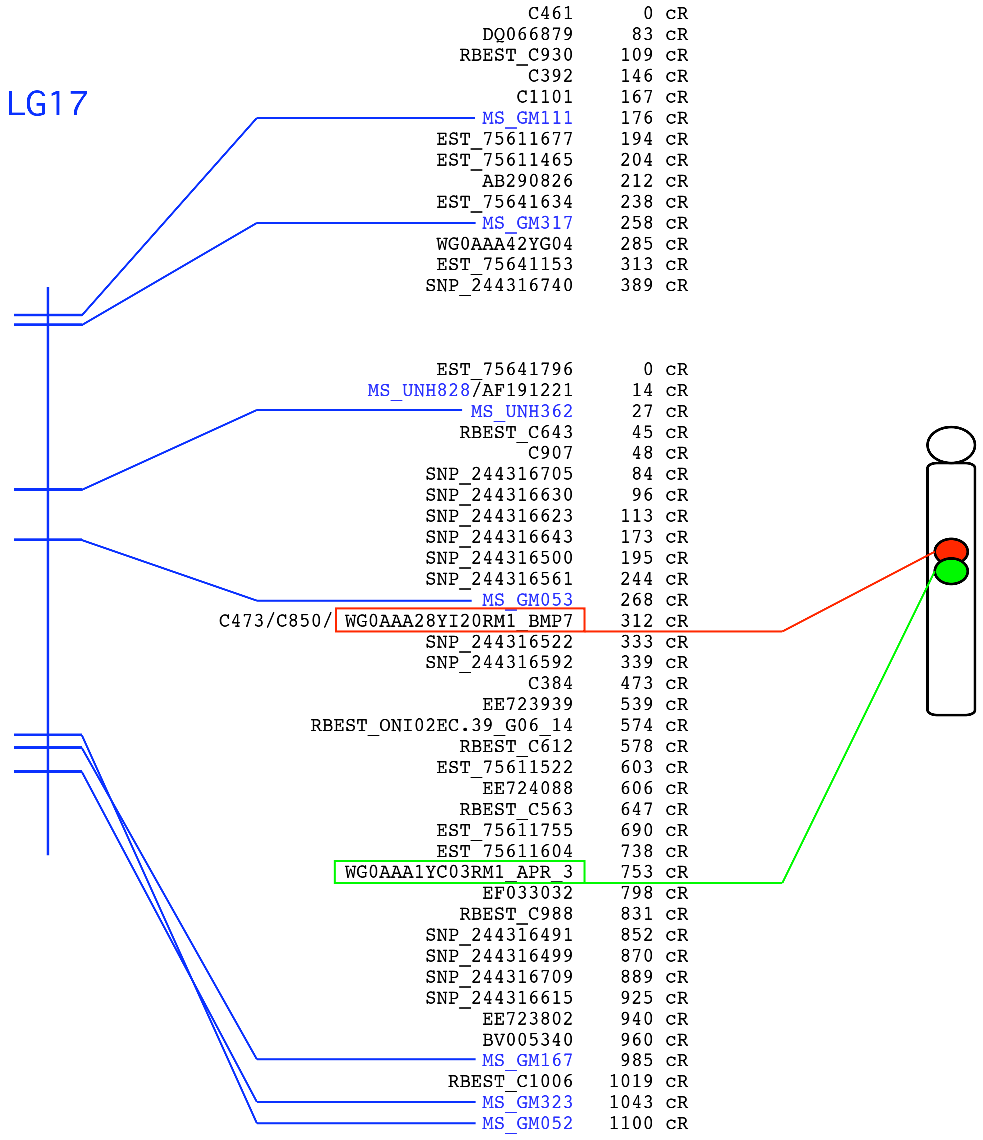
LG17
C461 0 cR
DQ066879 83 cR
RBEST_C930 109 cR
C392 146 cR
C1101 167 cR
MS_GM111 176 cR
EST_75611677 194 cR
EST_75611465 204 cR
AB290826 212 cR
EST_75641634 238 cR
MS_GM317 258 cR
WG0AAA42YG04 285 cR
EST_75641153 313 cR
SNP_244316740 389 cR
EST_75641796 0 cR
MS_UNH828/AF191221 14 cR
MS_UNH362 27 cR
RBEST_C643 45 cR
C907 48 cR
SNP_244316705 84 cR
SNP_244316630 96 cR
SNP_244316623 113 cR
SNP_244316643 173 cR
SNP_244316500 195 cR
SNP_244316561 244 cR
MS_GM053 268 cR
C473/C850/WG0AAA28YI20RM1_BMP7 312 cR
SNP_244316522 333 cR
SNP_244316592 339 cR
C384 473 cR
EE723939 539 cR
RBEST_ONI02EC.39_G06_14 574 cR
RBEST_C612 578 cR
EST_75611522 603 cR
EE724088 606 cR
RBEST_C563 647 cR
EST_75611755 690 cR
EST_75611604 738 cR
WG0AAA1YC03RM1_APR_3 753 cR
EF033032 798 cR
RBEST_C988 831 cR
SNP_244316491 852 cR
SNP_244316499 870 cR
SNP_244316709 889 cR
SNP_244316615 925 cR
EE723802 940 cR
BV005340 960 cR
MS_GM167 985 cR
RBEST_C1006 1019 cR
MS_GM323 1043 cR
MS_GM052 1100 cR

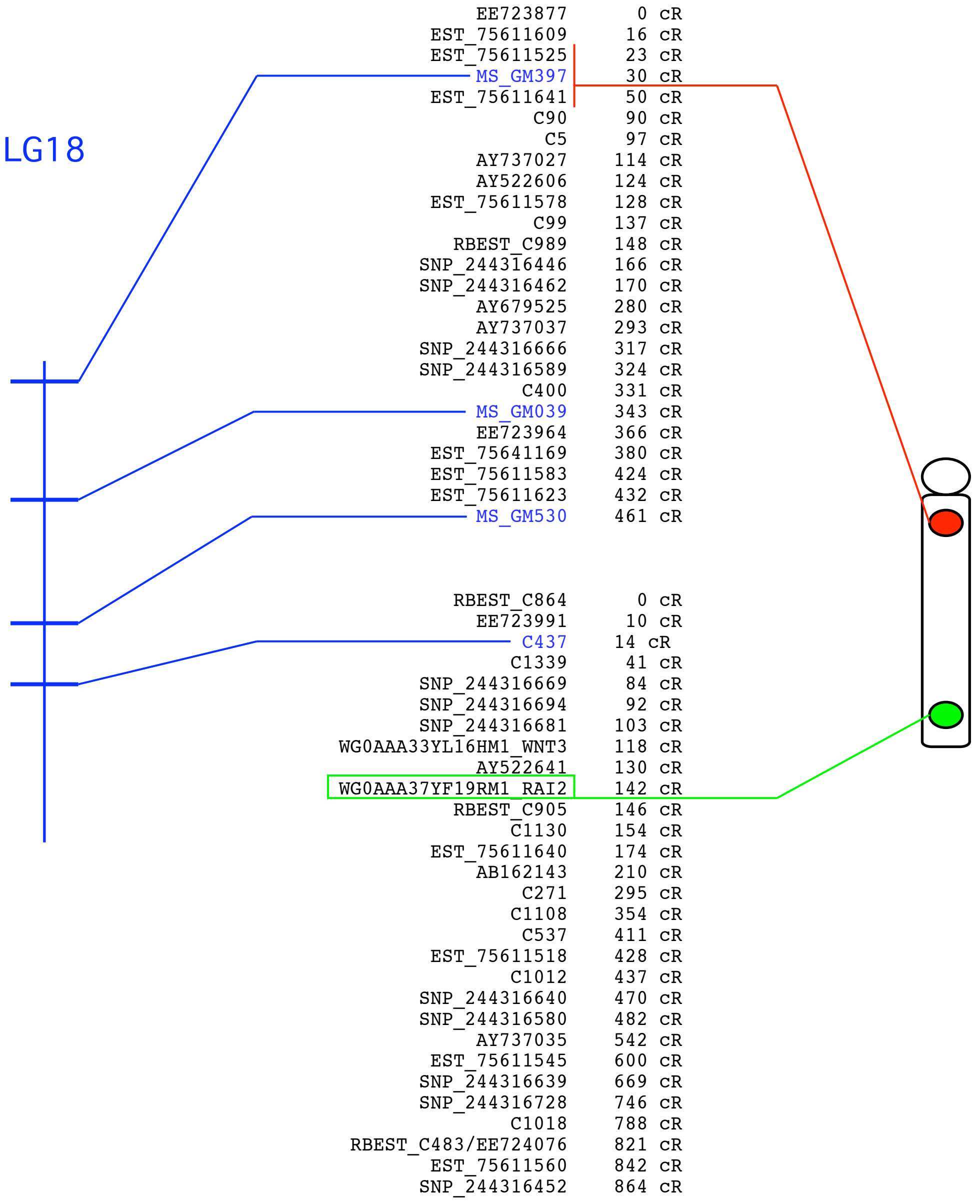

LG18
EE723877 0 cR
EST_75611609 16 cR
EST_75611525 23 cR
MS_GM397 30 cR
EST_75611641 50 cR
C90 90 cR
C5 97 cR
AY737027 114 cR
AY522606 124 cR
EST_75611578 128 cR
C99 137 cR
RBEST_C989 148 cR
SNP_244316446 166 cR
SNP_244316462 170 cR
AY679525 280 cR
AY737037 293 cR
SNP_244316666 317 cR
SNP_244316589 324 cR
C400 331 cR
MS_GM039 343 cR
EE723964 366 cR
EST_75641169 380 cR
EST_75611583 424 cR
EST_75611623 432 cR
MS_GM530 461 cR
RBEST_C864 0 cR
EE723991 10 cR
C437 14 cR
C1339 41 cR
SNP_244316669 84 cR
SNP_244316694 92 cR
SNP_244316681 103 cR
WG0AAA33YL16HM1_WNT3 118 cR
AY522641 130 cR
WG0AAA37YF19RM1_RAI2 142 cR
RBEST_C905 146 cR
C1130 154 cR
EST_75611640 174 cR
AB162143 210 cR
C271 295 cR
C1108 354 cR
C537 411 cR
EST_75611518 428 cR
C1012 437 cR
SNP_244316640 470 cR
SNP_244316580 482 cR
AY737035 542 cR
EST_75611545 600 cR
SNP_244316639 669 cR
SNP_244316728 746 cR
C1018 788 cR
RBEST_C483/EE724076 821 cR
EST_75611560 842 cR
SNP_244316452 864 cR

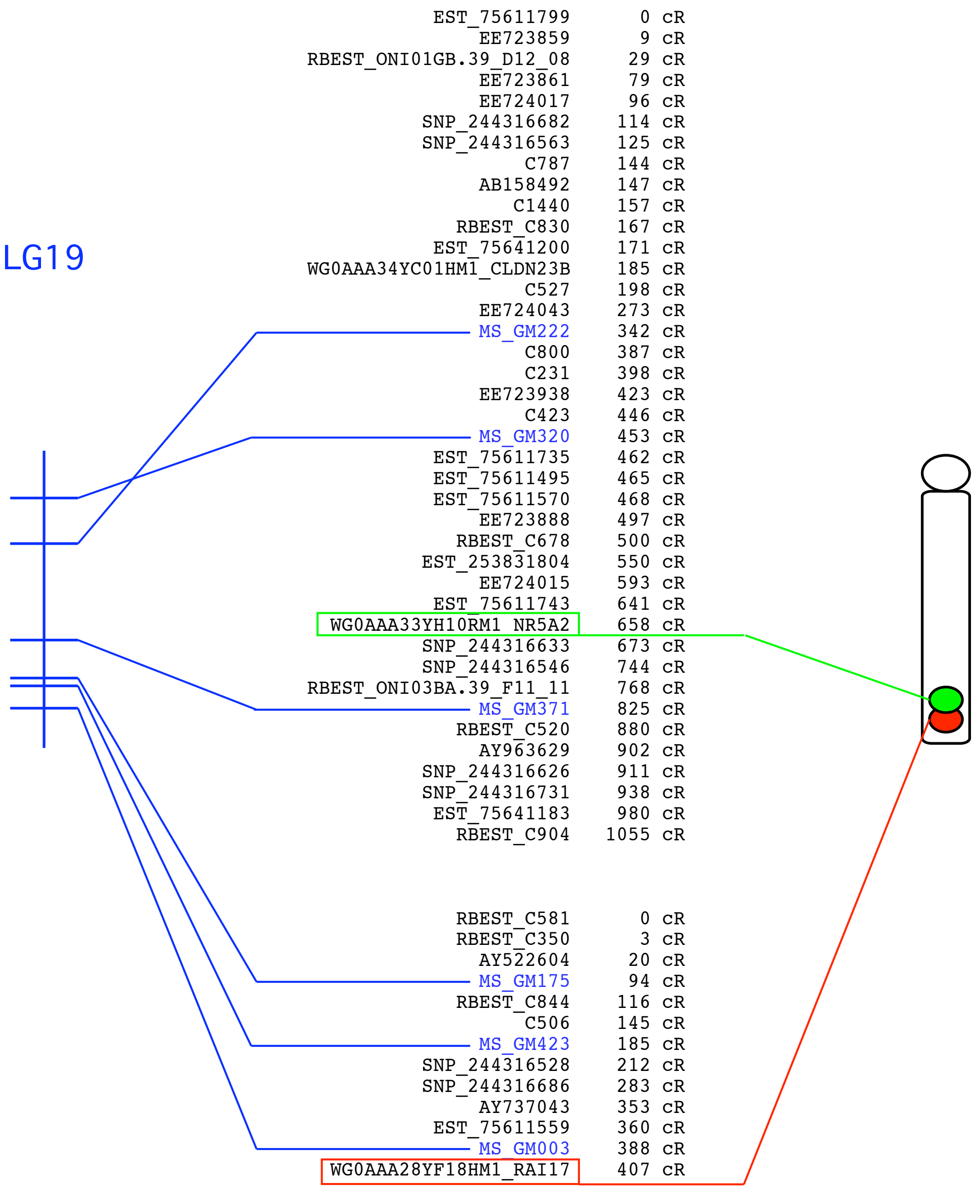
LG19
EST_75611799 0 cR
EE723859 9 cR
RBEST_ONI01GB.39_D12_08 29 cR
EE723861 79 cR
EE724017 96 cR
SNP_244316682 114 cR
SNP_244316563 125 cR
C787 144 cR
AB158492 147 cR
C1440 157 cR
RBEST_C830 167 cR
EST_75641200 171 cR
WG0AAA34YC01HM1_CLDN23B 185 cR
C527 198 cR
EE724043 273 cR
MS_GM222 342 cR
C800 387 cR
C231 398 cR
EE723938 423 cR
C423 446 cR
MS_GM320 453 cR
EST_75611735 462 cR
EST_75611495 465 cR
EST_75611570 468 cR
EE723888 497 cR
RBEST_C678 500 cR
EST_253831804 550 cR
EE724015 593 cR
EST_75611743 641 cR
WG0AAA33YH10RM1_NR5A2 658 cR
SNP_244316633 673 cR
SNP_244316546 744 cR
RBEST_ONI03BA.39_F11_11 768 cR
MS_GM371 825 cR
RBEST_C520 880 cR
AY963629 902 cR
SNP_244316626 911 cR
SNP_244316731 938 cR
EST_75641183 980 cR
RBEST_C904 1055 cR
RBEST_C581 0 cR
RBEST_C350 3 cR
AY522604 20 cR
MS_GM175 94 cR
RBEST_C844 116 cR
C506 145 cR
MS_GM423 185 cR
SNP_244316528 212 cR
SNP_244316686 283 cR
AY737043 353 cR
EST_75611559 360 cR
MS_GM003 388 cR
WG0AAA28YF18HM1_RAI17 407 cR

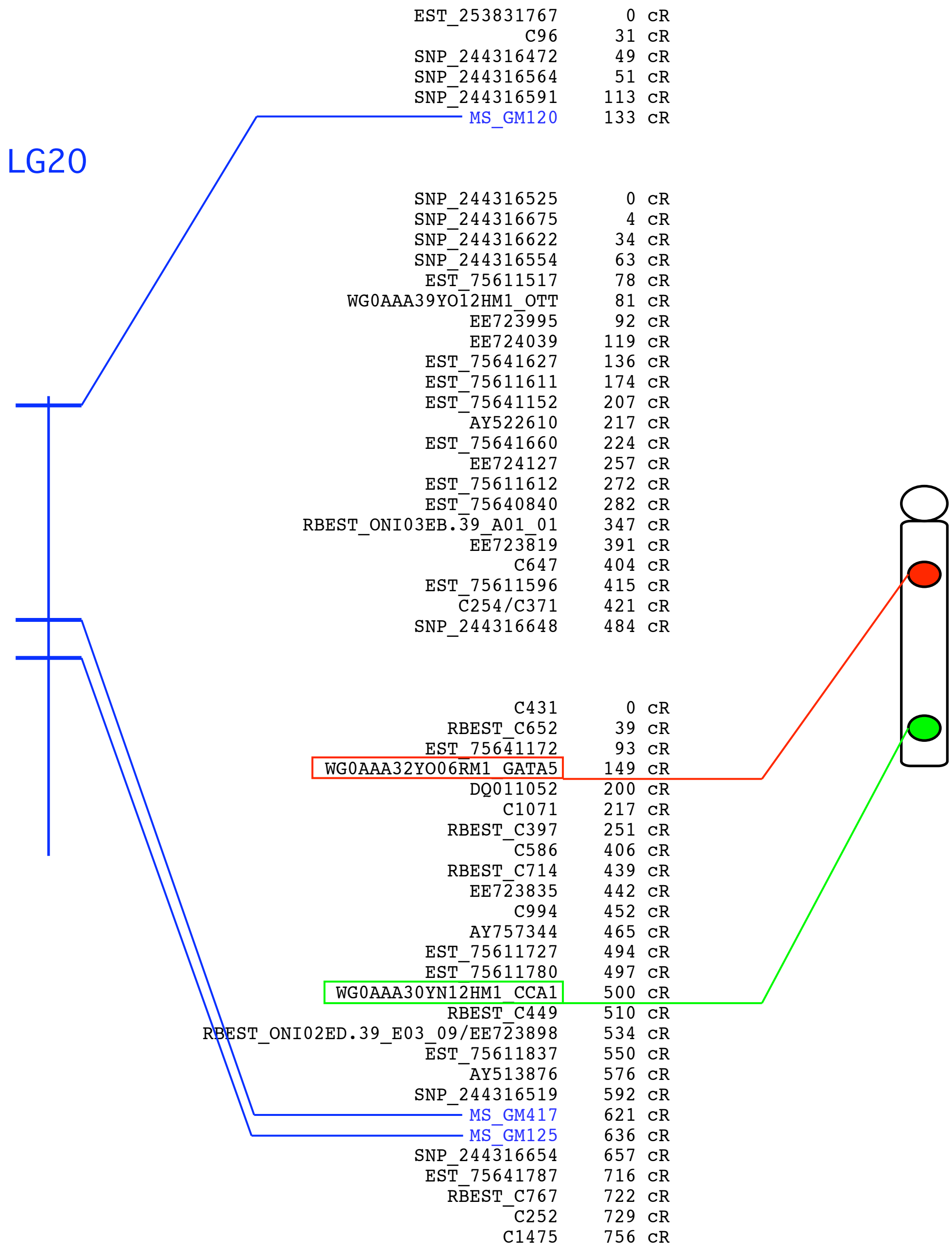
LG20
EST_253831767 0 cR
C96 31 cR
SNP_244316472 49 cR
SNP_244316564 51 cR
SNP_244316591 113 cR
MS_GM120 133 cR
SNP_244316525 0 cR
SNP_244316675 4 cR
SNP_244316622 34 cR
SNP_244316554 63 cR
EST_75611517 78 cR
WG0AAA39YO12HM1_OTT 81 cR
EE723995 92 cR
EE724039 119 cR
EST_75641627 136 cR
EST_75611611 174 cR
EST_75641152 207 cR
AY522610 217 cR
EST_75641660 224 cR
EE724127 257 cR
EST_75611612 272 cR
EST_75640840 282 cR
RBEST_ONI03EB.39_A01_01 347 cR
EE723819 391 cR
C647 404 cR
EST_75611596 415 cR
C254/C371 421 cR
SNP_244316648 484 cR
C431 0 cR
RBEST_C652 39 cR
EST_75641172 93 cR
WG0AAA32YO06RM1_GATA5 149 cR
DQ011052 200 cR
C1071 217 cR
RBEST_C397 251 cR
C586 406 cR
RBEST_C714 439 cR
EE723835 442 cR
C994 452 cR
AY757344 465 cR
EST_75611727 494 cR
EST_75611780 497 cR
WG0AAA30YN12HM1_CCA1 500 cR
RBEST_C449 510 cR
RBEST_ONI02ED.39_E03_09/EE723898 534 cR
EST_75611837 550 cR
AY513876 576 cR
SNP_244316519 592 cR
MS_GM417 621 cR
MS_GM125 636 cR
SNP_244316654 657 cR
EST_75641787 716 cR
RBEST_C767 722 cR
C252 729 cR
C1475 756 cR

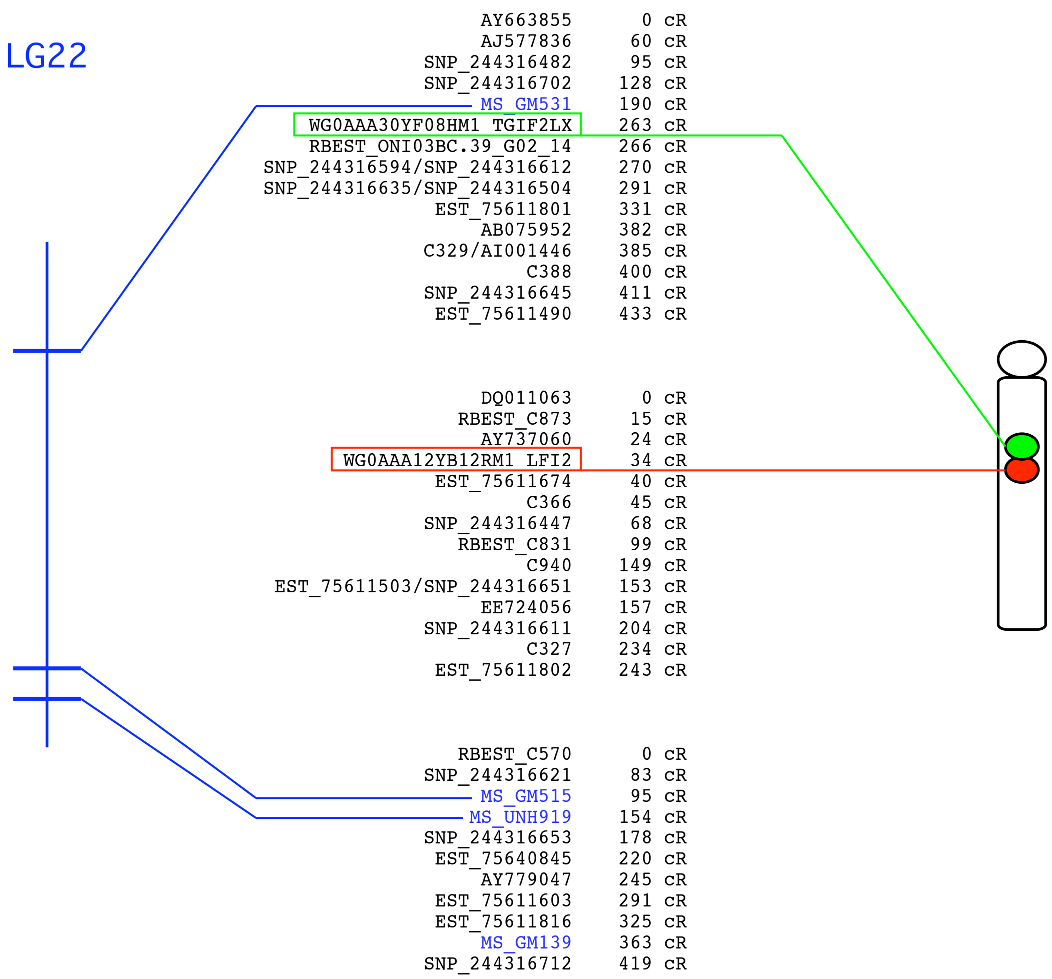

LG22
AY663855 0 cR
AJ577836 60 cR
SNP_244316482 95 cR
SNP_244316702 128 cR
MS_GM531 190 cR
WG0AAA30YF08HM1_TGIF2LX 263 cR
RBEST_ONI03BC.39_G02_14 266 cR
SNP_244316594/SNP_244316612 270 cR
SNP_244316635/SNP_244316504 291 cR
EST_75611801 331 cR
AB075952 382 cR
C329/AI001446 385 cR
C388 400 cR
SNP_244316645 411 cR
EST_75611490 433 cR
DQ011063 0 cR
RBEST_C873 15 cR
AY737060 24 cR
WG0AAA12YB12RM1_LFI2 34 cR
EST_75611674 40 cR
C366 45 cR
SNP_244316447 68 cR
RBEST_C831 99 cR
C940 149 cR
EST_75611503/SNP_244316651 153 cR
EE724056 157 cR
SNP_244316611 204 cR
C327 234 cR
EST_75611802 243 cR
RBEST_C570 0 cR
SNP_244316621 83 cR
MS_GM515 95 cR
MS_UNH919 154 cR
SNP_244316653 178 cR
EST_75640845 220 cR
AY779047 245 cR
EST_75611603 291 cR
EST_75611816 325 cR
MS_GM139 363 cR
SNP_244316712 419 cR

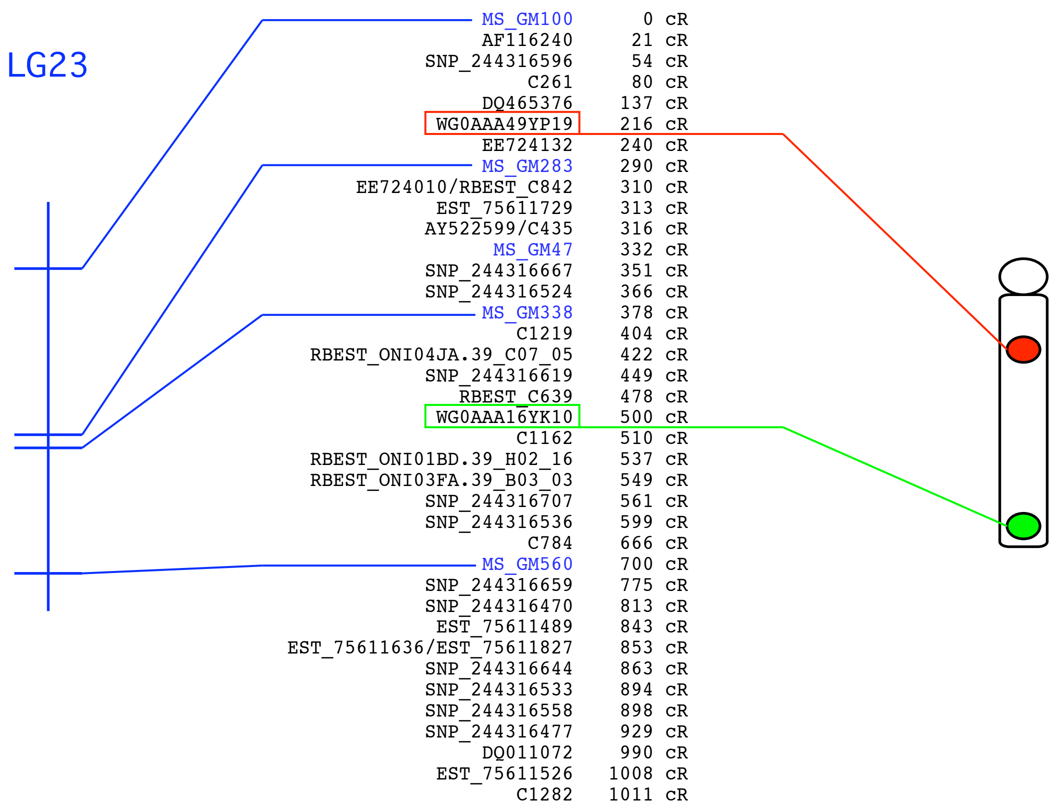
LG23
MS_GM100 0 cR
AF116240 21 cR
SNP_244316596 54 cR
C261 80 cR
DQ465376 137 cR
WG0AAA49YP19 216 cR
EE724132 240 cR
MS_GM283 290 cR
EE724010/RBEST_C842 310 cR
EST_75611729 313 cR
AY522599/C435 316 cR
MS_GM47 332 cR
SNP_244316667 351 cR
SNP_244316524 366 cR
MS_GM338 378 cR
C1219 404 cR
RBEST_ONI04JA.39_C07_05 422 cR
SNP_244316619 449 cR
RBEST_C639 478 cR
WG0AAA16YK10 500 cR
C1162 510 cR
RBEST_ONI01BD.39_H02_16 537 cR
RBEST_ONI03FA.39_B03_03 549 cR
SNP_244316707 561 cR
SNP_244316536 599 cR
C784 666 cR
MS_GM560 700 cR
SNP_244316659 775 cR
SNP_244316470 813 cR
EST_75611489 843 cR
EST_75611636/EST_75611827 853 cR
SNP_244316644 863 cR
SNP_244316533 894 cR
SNP_244316558 898 cR
SNP_244316477 929 cR
DQ011072 990 cR
EST_75611526 1008 cR
C1282 1011 cR
